# Environmental Insights and Sustainability Opportunities for Scaled‐Up MXene Production Without Etching

**DOI:** 10.1002/advs.75376

**Published:** 2026-04-27

**Authors:** Yushuai Huang, Peng Peng, Maoqiao Xiang, Fen Yue, Jiangyan Wang, Wenwei Liu, Qingshan Zhu

**Affiliations:** ^1^ State Key Laboratory of Mesoscience and Process Engineering Institute of Process Engineering Chinese Academy of Sciences Beijing China; ^2^ School of Chemical Engineering University of Chinese Academy of Sciences Beijing China; ^3^ State Key Laboratory of Biopharmaceutical Preparation and Delivery Institute of Process Engineering, Chinese Academy of Sciences Beijing China

**Keywords:** carbon emission, environmental impacts, MXene, process‐based life cycle assessment, sustainable synthesis

## Abstract

MXenes are a class of high‐performance materials receiving significant attention in areas such as energy and sensors. However, their conventional synthesis via defective etching raises environmental concerns due to the heavy use of hazardous chemicals. Herein, the environmental impacts of emerging MXene synthesis pathways are systematically evaluated from lab to process scales. Transforming away from solvent‐based etching could enable a 94% reduction in carbon emissions with improved reactant efficiency and recovery. Further, process optimization opportunities are identified for gas‐phase and molten salt synthesis pathways to achieving 67% to 85% reduction in human health, ecosystems, and natural resource impacts. Future research should target developing processes with dispersant to product ratio reaching 5.3 L/kg, which can reduce the carbon emission of MXene production down to 27 kg CO_2_‐eq/kg, making it one of the leading advanced nanomaterials.

## Graphical abstract

Through a systematic and comprehensive analysis of the environmental impacts for the emerging MXene synthesis pathways, this study presents process transformation and optimization opportunities for low‐carbon MXene production from laboratory to industrial scales.

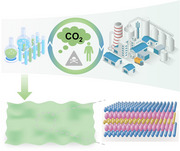

## 1 Introduction

Developing advanced materials has been viewed as essential for achieving various decarbonization goals [1, 2, 3]. Within the past decade, the number and amount of research and development related to nano‐scale high‐performing materials have greatly emerged [4]. Since their first synthesis [5], MXenes, usually denoted as M_n+1_X_n_T_x_ (n = 1–4), have received significant attention as a potentially disruptive class of advanced material owing to their unique chemical structure and composition [6, 7, 8, 9]. These materials are constructed from two or more transition metal (M) layers arranged in a hexagonal close‐packed configuration, where the X (carbon or nitrogen) atoms occupy the octahedral sites between adjacent M layers, with the surface terminated by single or mixed functional groups (T) [9, 10]. Previous studies have demonstrated that these structural features enable superior physical and electrochemical properties [11, 12, 13], supporting their applications in energy [14, 15, 16], sensors [17, 18, 19], electromagnetic interference shielding [20, 21, 22], and biomedicine [23, 24, 25].

Despite their performance, MXenes themselves are typically synthesized through chemical or electrochemical etching of the A‐layer atoms (such as Al, Si, Ga) from MAX precursors (M_n+1_AX_n_) [9]. Although this is a relatively mature process, it often requires the use of hydrofluoric acid [26], which induces cationic defects that reduces environmental stability [27], and generates mixed functional groups [13]. As hydrofluoric acid is highly toxic and corrosive [28], the wastewater stream from MXenes production is hazardous, requiring significant post treatment [29, 30]. Other emerging etching methods include NH_4_HF_2_ low‐temperature molten salt (LTMS) and chemical scissoring (CS), which rely on melts with relatively high fluidily to accelerate etchant penetration and utilize Lewis acid as redox scissors to selectively remove A‐layer atoms, respectively [31, 32]. Together with the use of MAX precursors, MXene production by etching has carbon emissions up to over 428 kg CO_2_‐eq/kg [30, 33].

To address these concerns, a direct synthesis method has been developed, which eliminates the reliance on MAX precursors etching by employing Ti and TiCl_4_ for MXene synthesis [6]. Ideally, using TiCl_4_ to produce MXene is cheaper as pure Ti is prepared via reducing Ti oxides or salts. However, this method, regardless of the use of precursor, inevitably introduces impurities, as the MXene product tends to cover the reactant source and create mass transport barriers during gas‐solid reactions [6, 34]. Following upon this, the recently developed gas‐phase synthesis method for preparing pure Cl‐terminated Ti_2_CCl_2_ MXenes is designed based on advanced fluidized reaction systems [34, 35]. It is notable that due to its potential for continuous and scalable production, this method also demonstrates a certain level of commercialization feasibility. However, the environmental sustainability of the above‐mentioned emerging MXene synthesis methods remains to be systematically explored, as current life cycle assessment (LCA) around MXenes have been focusing on the laboratory‐scale chemical etching methods for Ti_3_C_2_T_x_ MXenes [33, 36, 37, 38].

**FIGURE 1:**
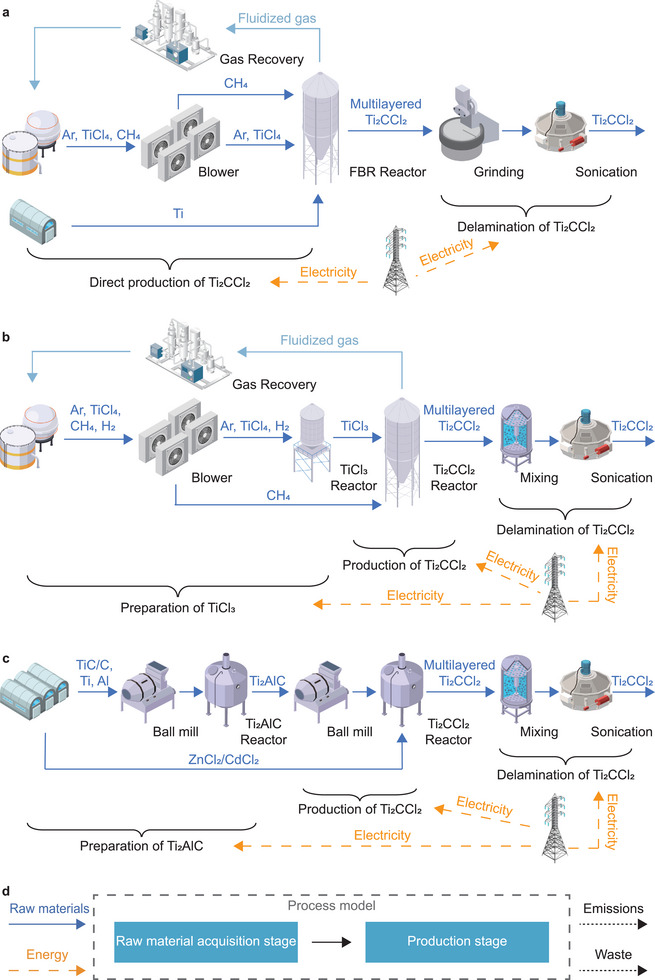
Simplified diagram of the four Ti_2_CCl_2_ production processes. (a) In‐situ gas‐phase synthesis. (b) Multi‐stage gas‐phase synthesis. (c) The process for molten‐ZnCl_2_ and molten‐CdCl_2_ synthesis. (d) The ‘from cradle to gate’ system boundary for using LCA to evaluate the four processes. Detailed processes are included in Figures S1–S4.

To address these knowledge gaps, we employ rigorous process design and modelling to offer scale‐up implications, and use LCA to comprehensively analyze the environmental impacts across the existing and emerging MXene production pathways at the process level (Figure 1 and Methods). Beyond assessing its carbon footprint as the primary LCA performance indicator, we also evaluate other environmental impacts including the effects on human health and natural resources. Sensitivity analysis shows the quantitative improvement potential in process optimization versus process transformation for key factors such as gas recovery rate and unit dispersant usage. As such, findings from this study are anticipated to offer guidance for advancing the functional promises and environmental friendliness of the future industrialized production of MXenes, further advancing our efforts towards deep decarbonization.

## 2 Discussion

### 2.1 Cross Comparison of Carbon Emissions and Breakdowns

**FIGURE 2:**
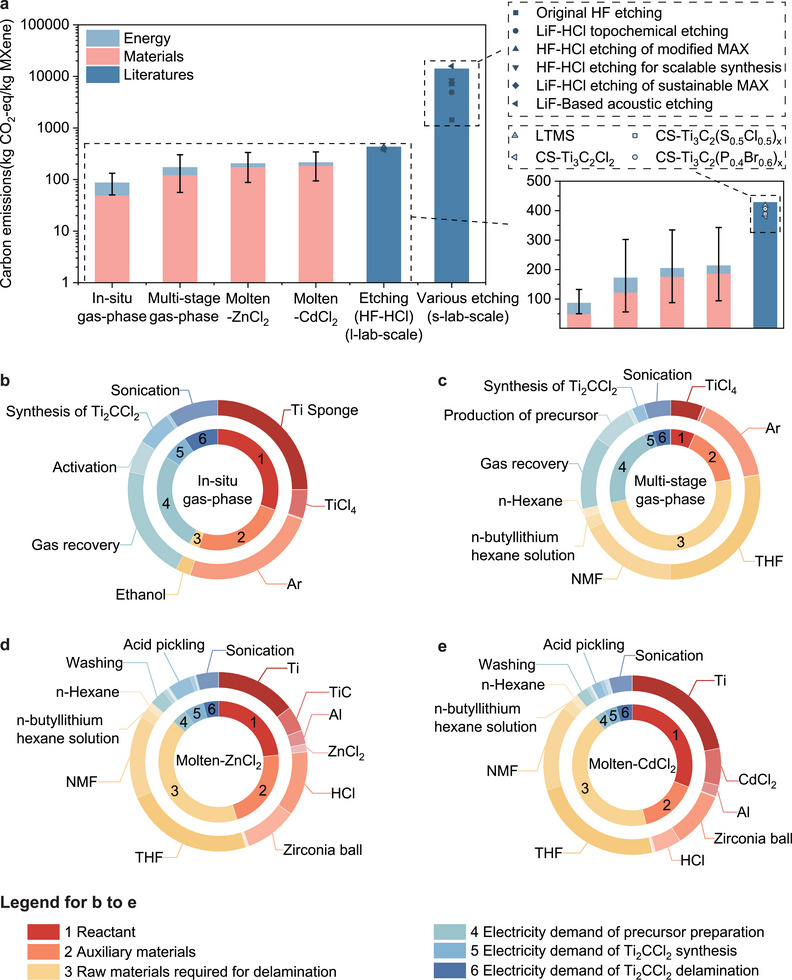
Schematic overview of carbon emissions across four synthesis methods. (a) Comparative carbon emissions results. The conventional etching methods are derived from [5, 33, 38, 43, 44, 45, 46, 47]. (b–e) Carbon emissions contributions from processes and materials of four synthesis methods.

In this study, the MXene synthesis pathways are generally categorized in Figure 1a–c as in‐situ gas‐phase synthesis, multi‐stage gas‐phase synthesis, and molten synthesis, respectively. These pathways differ from each other in terms of synthesis environment (gas vs. aqueous) and the use of pre‐cursors (Zn vs. Cd within the molten synthesis). Detailed descriptions of these methods and their key process parameters are included in Methods and Notes S1–S4. Beyond cross comparison within these Ti_2_CCl_2_ synthesis pathways, they are further compared with the etching methods using HF‐HCl or LiF‐HCl, LTMS method and CS method. Based on literature availability, the MXenes analyzed for the conventional etching and LTMS methods are Ti_3_C_2_T_x_, and those for the CS method are Ti_3_C_2_Cl_2_, Ti_3_C_2_(S_0.5_Cl_0.5_)_x_ and Ti_3_C_2_(P_0.4_Br_0.6_)_x_ [31, 32, 33, 38]. Figure 2a shows that among the synthesis methods considered in this study, the in‐situ gas‐phase synthesis method has the lowest carbon emissions. As previously mentioned (Introduction section), this is primarily because the etching methods necessitate the MAX precursors and involve carbon‐intensive chemicals, coupled with the environmental burden from toxic by‐product disposal [30, 33, 38]. For instance, while the emerging LTMS method enables high etching speed at low temperature, the excessive reaction molar ratio of NH_4_HF_2_ to Ti_3_AlC_2_ (20:1) limits its potential for carbon emission reduction. Collectively, the carbon emissions for four main synthesis methods are in‐situ gas‐phase (86 kg CO_2_‐eq/kg MXene), multi‐stage gas‐phase (172 kg CO_2_‐eq/kg MXene), molten‐ZnCl_2_ (204 kg CO_2_‐eq/kg MXene), molten‐CdCl_2_ (213 kg CO_2_‐eq/kg MXene), and details are included in the Methods section.

To systematically explore the driving forces of the above carbon emission results, and to elucidate the environmental impact variations across processing stages, we comparatively analyze the distribution of carbon emissions (Figure 2b‐e). These distributions reflect the emission contributions from raw material acquisition and the energy consumption in MXene production stages for four synthesis methods. Notably, although the in‐situ gas‐phase synthesis method has the lowest overall carbon emission, its reactant and auxiliary material usage in the raw material acquisition stage accounts for the largest proportion (55%). On the other hand, the multi‐stage gas‐phase synthesis has a relatively more complex process, yet its overall conversion rate is lower than the in‐situ gas‐phase synthesis (48% vs 90%, respectively). As such, the multi‐stage gas‐phase method has a higher carbon emission related to the use of Ar and TiCl_4_, which persists even with the presence of an integrated recovery design (see the Sensitivities section for details). For the molten salt pathways, they collectively demonstrate higher proportions of carbon emissions from reactant and auxiliary material usage (45% and 46% for the two molten salt methods). The main difference between the molten salt methods from the gas‐phase methods is the involvement of producing the complex MAX phase prior to MXene formation. Replacing the MAX phase with a simpler precursor of TiCl_3_, as achieved by the gas‐phased methods, is beneficial in terms of reducing the overall carbon emissions.

In addition, note that except for the in‐situ gas‐phase synthesis method, all other three methods exhibit significantly higher carbon emissions from raw material consumption in the delamination, which are 49%, 41%, and 43% for the multi‐stage gas‐phase synthesis method, molten‐ZnCl_2_, and molten‐CdCl_2_, respectively. And the breakdowns in Figure 2c‐e indicates that the use of relatively more complex organic solvents (e.g. THF, NMF, and n‐butyllithium hexane solution, compared to ethanol) to treat MXene in the Ti_2_CCl_2_ for delamination is another cause of high carbon emissions.

Besides material usage, the carbon emissions resulting from energy consumption (see the Methods section for its detailed definition and boundary) during the production stage account for higher portions in the gas‐phase synthesis methods (21% and 12% for cryogenic distillation [39] and electrothermal component in the in‐situ gas‐phase, respectively due to gas recovery and heating) compared to 0.1–2% and 0.1–1% for molten‐ZnCl_2_ and molten‐CdCl_2_ methods. These differences primarily stem from the need of argon inert gas consumption with high flow rate, and the energy consumption to compensate the heat loss in the gas‐phase synthesis methods. Therefore, increasing the efficiency of gas utilization and process heating would be the key improvements required for the gas‐phase synthesis methods. Specifically, on the basis of ensuring a stable supply of TiCl_4_, the excessive gas demand could be reduced by process design such as optimizing internal gas distributors for mixing uniformity and incorporating internal baffles to regulate the gas flow path [40, 41]. For context, if the Ar:CH_4_ mole ratio is safely reduced by 50% (from 4:1 to 2:1), the energy‐related carbon emissions would decrease by 30% for the in‐situ gas‐phase synthesis (Figure S5). Furthermore, introducing advanced insulation materials [42] to reduce the thermal conductivity by 50% would further decrease these emissions by 5%.

It is notable that all four methods exhibit high carbon emissions during the sonication process in the delamination stage, as it is a relatively energy‐intensive step. Interestingly, for the LiF‐HCl etching method, Li^+^ is spontaneously inserted between the MXene layers during etching, while the multi‐stage gas‐phase, molten‐ZnCl_2_ and molten‐CdCl_2_ methods require complex delamination steps. This inherent advantage allows the LiF‐HCl method to avoid the demand of both extra carbon‐intensive organic delamination reagent and sonication process [26, 38]. And the in‐situ gas‐phase method also achieves a simplified delamination process by directly producing a product with three‐dimensional flower‐like structure [35]. Consequently, although the LiF‐HCl method exhibts higher total carbon emissions, its carbon emissions of delamination are comparable to the in‐situ gas‐phase method, and are lower than those of the other three methods (Figure S6). Therefore, for synthesis methods that require additional delamination steps, improvements in developing more efficient delamination processes (see the Insights to sustainable commercialization section for details) for MXene production would be an important future research direction in the field.

### 2.2 Sensitivities

**FIGURE 3:**
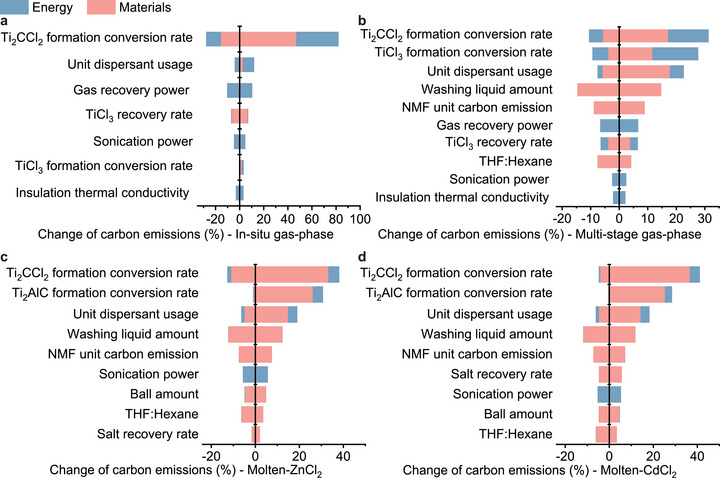
Sensitivity analysis results for the four MXene synthesis methods (a) in‐situ gas‐phase, (b) multi‐stage gas‐phase (c) molten‐ZnCl_2_ (d) molten‐CdCl_2_. Note that the effect of gas recovery rate of the gas‐phase methods is not plotted as its increase leads to higher energy‐related emission, but lower chemical‐related emission. Instead, its impact is studied in detail later in Figure 4.

In order to inform future process optimization strategies, sensitivity analysis is performed by systematically varying selected key process parameters within a ±50% variation range or other reasonable ranges based on actual process design (Figure 3, Methods, and Tables S1–S4). For all four synthesis methods, attention should be paied to the effects of conversion rates for both MXene formation and precursor formation. Quantitatively, reducing 50% of conversion rate for Ti_2_CCl_2_ formation can lead to over 80% increase in carbon emissions for the in‐situ gas‐phase method, compared to 31%, 38% and 41% for the multi‐stage gas‐phase, molten‐ZnCl_2,_ and molten‐CdCl_2_ methods. This is primarily attributed to the dual burden imposed by a decrease in conversion at the same production scale, which amplified the demands associated with both the feedstock and the auxiliary materials required for the operation. This is evident in the in‐situ gas‐phase method, where the increased reactant methane load leads to the proportional increase in auxiliary Ar usage, with Ar‐related emissions accounting for 55% of the total variation.

Between the two gas‐phase synthesis methods, their distinctions are primarily the different reactants used in the precursor preparation stage, which subsequently effects the conversion rates from TiCl_4_ to TiCl_3_. For the in‐situ gas‐phase method, a 50% reduction in the conversion rate of TiCl_4_ to TiCl_3_ in the precursor preparation increases the total carbon emissions by 3%, while for the multi‐stage gas‐phase method, the increase is 27% (and 16% of this is from energy consumption). This difference is mainly due to the fact that the multi‐stage gas‐phase method exclusively rely on TiCl_4_ as titanium source for TiCl_3_. So, the conversion rate has a higher impact on the reactant gas consumption and the required reactor volume. Whereas for the in‐situ gas‐phase method, a titanium sponge is also involved in the reaction to produce TiCl_3_, which somewhat reduces the reliance of converting TiCl_4_. For the two molten salt methods, their conversion rates of the precursor formation have greater influences on the carbon emissions compared to the gas‐phase synthesis methods. Specifically, the increase in carbon emission of the molten‐ZnCl_2_ method is 31%, which is resulted from 50% reductions in conversion rates of Ti_2_AlC formations, while the increase of the molten‐CdCl_2_ method based on the effect of conversion rate is 28%. Therefore, enhancing conversion rate should be the emphasis for future MXene syntheses. This could be achieved through process intensification and reactor optimization, for example to enhance the mass transfer between reactants.

Furthermore, not only does the delamination process account for a substantial portion of carbon emissions, its relevant operational parameters also significantly influence the overall emissions. In the sensitivity analysis, this is quantified as the unit dispersant usage, which is mostly ethanol for the in‐situ gas‐phase method and NMF for the other three methods. Other than the dispersants, the energy consumption associated with recovery of the carrying gas and reactants, as well as the amount of washing liquid used, are all critical parameters, and can be improved via more efficient process designs.

### 2.3 Insights to Sustainable Commercialization

**FIGURE 4:**
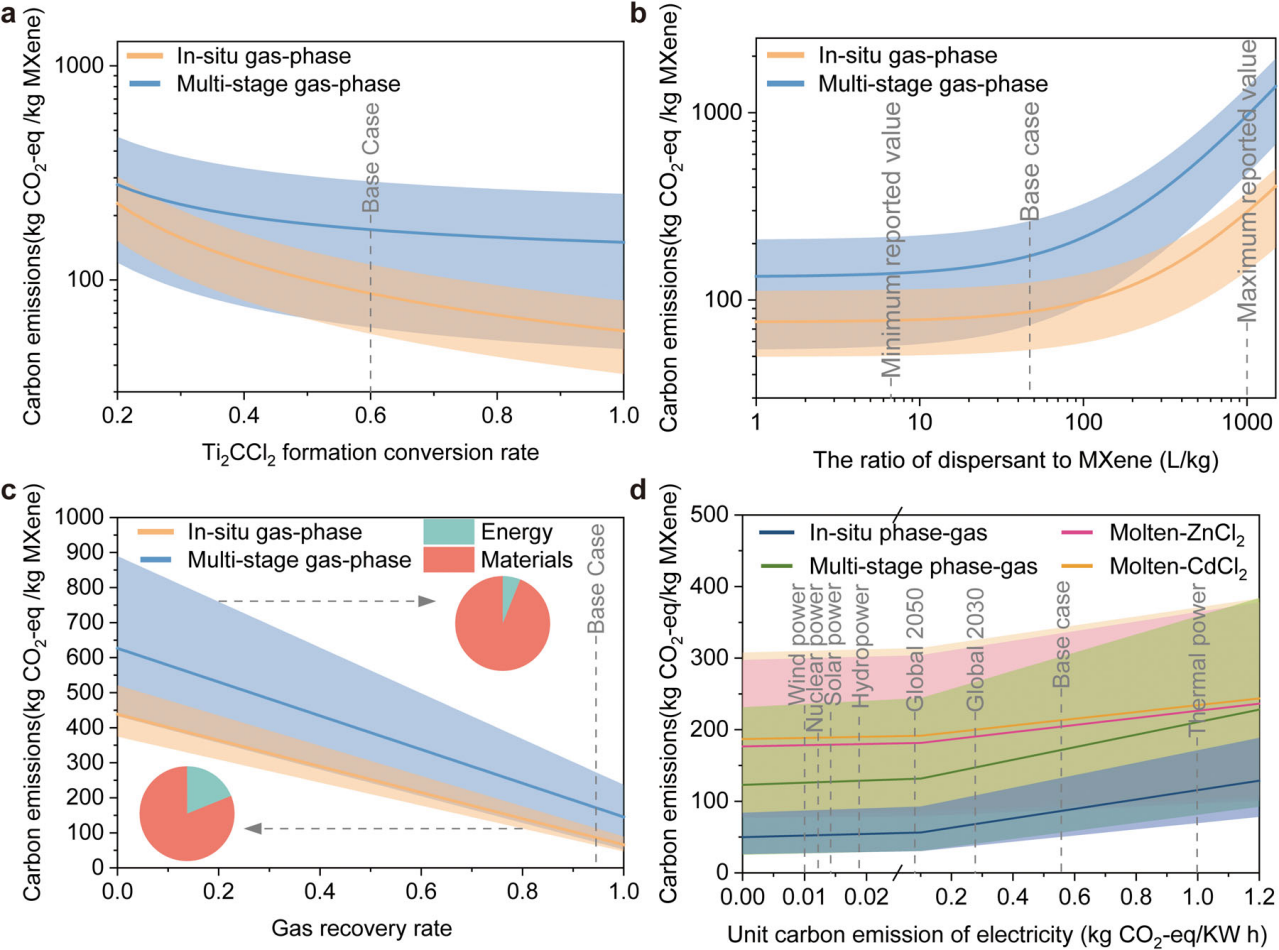
The effects of various key process parameters to the carbon footprint of MXene. (a) the Ti_2_CCl_2_ formation conversion rate, (b) the ratio of dispersant to MXene (ethanol for the in‐situ gas‐phase method, NMF for the multi‐stage gas‐phase method), (c) the gas recovery rate, (d) the unit carbon emission factor of electricity.

Sensitivity analysis shows that Ti_2_CCl_2_ formation conversion rate is the most critical parameter influencing the carbon emissions of MXene production. Despite the efficient transformation into Ti_2_CCl_2_ enabled by the high reactivity of TiCl_3_, a conservative conversion rate of 60% is adopted for the base case to ensure a cautious assessment [6, 34]. Maintaining a high conversion rate is essential in future scale‐up production. Specifically, improving the conversion rate from 20% to 100% drives a substantial emissions reduction of 75% (from 228 kg CO_2_‐eq/kg to 58 kg CO_2_‐eq/kg) for the in‐situ gas‐phase method, and a corresponding 46% reduction (from 279 kg CO_2_‐eq/kg to 150 kg CO_2_‐eq/kg) for the multi‐stage gas‐phase method (Figure 4a). Such improvement could be achieved not only through exploring alternativereaction pathways [6, 34, 35], but also via optimization of reaction conditions [48].

Besides the chemical conversions, the dispersant usage during delamination also contributes substantially to carbon emissions for MXene synthesis. Typically, the dispersant used in the MXene dispersion system, ethanol or NMF, has concentration range of 0.001–0.15 kg/L, which corresponds to dispersant to MXene ratios from 6.67 to 1000 L/kg (before introducing recovery or process optimization in Figure 3) [7, 34, 49, 50]. Our detailed analysis in Figure 4b shows that even within this regime, operating from the least to the most ideal value can lead to an 74% drop in carbon emission (from 295 kg CO_2_‐eq/kg to 78 kg CO_2_‐eq/kg) for the in‐situ gas‐phase method, and an 86% drop (from 965 kg CO_2_‐eq/kg to 138 kg CO_2_‐eq/kg) for the multi‐stage gas‐phase method. Therefore, it is imperative to reduce dispersant usage while maintaining system stability. During the scale‐up process, recommendations on achieving this would include the use of efficient shear force delamination, hydrodynamic‐assisted exfoliation, and high‐temperature ultrasound treatment [51, 52, 53]. These emerging delamination strategies utilize commercially‐available equipment, and theoretically can enable more scalable and high‐yield manufacturing, offering a more sustainable path with high industrial readiness.

Additionally, increasing the gas recovery rate of the gas‐phase synthesis methods also has significant benefit on improving the carbon emission of MXene produced. During the scale up, optimizing the gas recovery rate is relatively more straightforward comparing with other improvements, such as increasing the conversion rates. The latter typically requires complex reaction condition design or changing the reaction schemes. The optimization of gas recovery rate can be achieved via recycling through pressure swing adsorption and membrane separation technologies [54, 55], therefore, should be prioritized for scaling MXene production in the near future. For example, operating under 50% gas recovery rate would lead to 80% increase in carbon emission compared to 80% recovery rate for the in‐situ gas‐phase method (Figure 4c).

Other than the process parameters, the energy sources can also impact the sustainability of large‐scale MXene production in industrial settings. Figure 4d shows that under the base case scenario, the slopes between the changes in MXene emission and kg CO_2_/kWh electricity used are 65 (in‐situ gas‐phase), 88 (multi‐stage gas‐phase), 50 (molten‐ZnCl_2_), and 47 (molten‐CdCl_2_) for the four methods. Further, we highlight, using dashed lines, the specific power emission factors for different sources, and the projected global average values for 2030 and 2050 [56, 57]. The results imply that if coupled with renewable energy, the carbon emissions from MXene production would be comparable to those inherently generated during raw material acquisition. And the overall carbon emissions of MXenes can reach 50 kg CO_2_‐eq/kg to 187 kg CO_2_‐eq/kg within the four synthesis methods (with the lowest still being the in‐situ gas‐phase method) if solely powered by renewable electricity.

**FIGURE 5:**
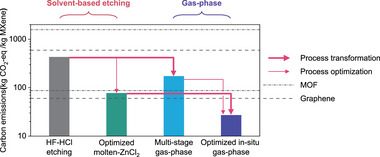
Carbon emissions of un‐optimized vs. optimized MXene production and comparison with other advanced materials. Carbon footprint of selected MOF and graphene production pathways obtained from literatures [58, 59].

Further, if all the above improvements (dispersant usage, gas recovery rate, renewable coupling, etc.) are achieved, the carbon emissions of MXene production can be reduced to 27 kg CO_2_‐eq/kg for the in‐situ gas‐phase synthesis (the lowest value in Figure 5). This result is 94% of reduction compared with the conventional solvent‐based etching using HF‐HCl (first column), and is lower than the reported values of other emerging high‐performing materials such as MOFs and graphene (87 to 1571 kg CO_2_‐eq/kg, and 60 to 594 kg CO_2_‐eq/kg, respectively) [58, 59]. This shows the need of developing transformative pathways (from solvent‐based etching to gas‐phase synthesis) and process optimization within gas‐phase synthesis when it comes to commercializing MXene productions. Even just by changing from solvent‐based etching using HF‐HCl to molten salt, the carbon emission value for MXene production would be 77 kg CO_2_‐eq/kg after improving the conversion rate and mixing agent recovery rate, which is still comparable with the selected representative MOFs and graphene production pathways.

**FIGURE 6:**
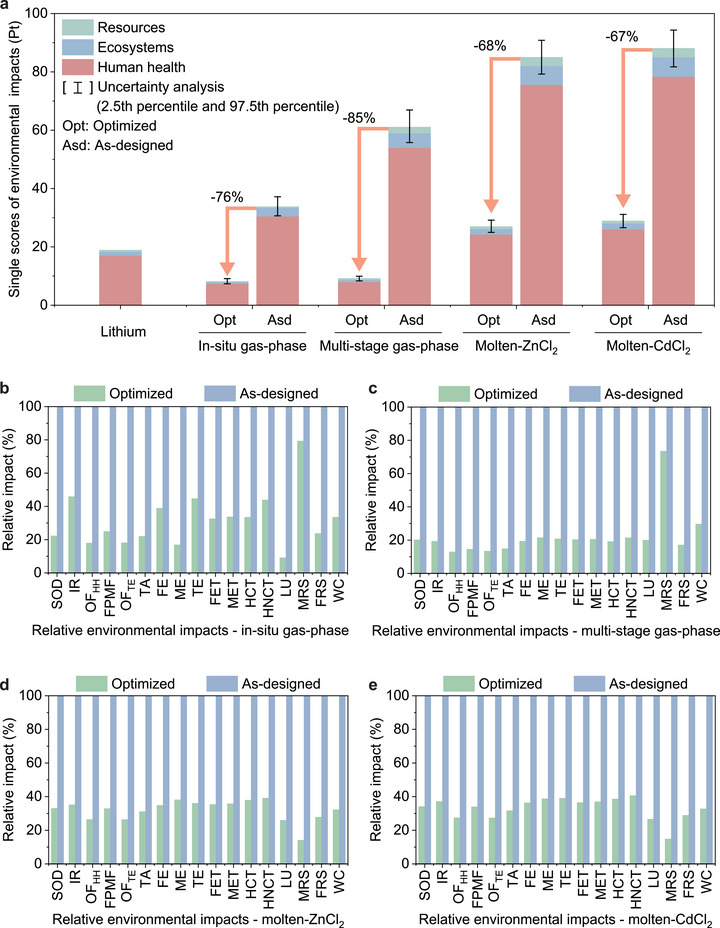
The environmental impacts analysis of as‐designed vs. optimized MXene production of the four synthesis methods under the Recipe 2016 at endpoint and midpoint levels. (a) Single scores of environmental impacts at the endpoint level. The lithium metal serves as a reference for comparative analysis with MXene. Error bars represent uncertainty results from Monte Carlo simulations (numerical results in Table S5). (b to e) Relative environmental impacts at the midpoint level. The impact categories are stratospheric ozone depletion (SOD, kg CFC‐11‐eq), ionizing radiation (IR, kBq Co‐60‐eq), ozone formation‐human health (OF_HH_, kg NO_x_‐eq), fine particulate matter formation (FPMF, kg PM2.5‐eq), ozone formation‐terrestrial ecosystems (OF_TE_, kg NO_x_‐eq), terrestrial acidification (TA, kg SO_2_ eq), freshwater eutrophication (FE, kg P‐eq), marine eutrophication (ME, kg N‐eq), terrestrial ecotoxicity (TE, kg 1,4‐DCB), freshwater ecotoxicity (FET, kg 1,4‐DCB), marine ecotoxicity (MET, kg 1,4‐DCB), human carcinogenic toxicity (HCT, kg 1,4‐DCB), human noncarcinogenic toxicity (HNCT, kg 1,4‐DCB), land use (LU, m^2^a crop‐eq), mineral resource scarcity (MRS, kg Cu‐eq), fossil resource scarcity (FRS, kg oil‐eq), and water consumption (WC, m^3^).

It is important to note that the environmental impact indicators are not limited to carbon emissions. Compared with lithium, a relatively more‐common industrial material, the environmental impact of MXene produced by as‐designed processes is 1.8 to 4.7 times higher (from in‐situ gas‐phase to molten‐CdCl_2,_ Figure 6a). However, after optimization, the environmental impacts of MXene produced via gas‐phase or molten salt methods can be reduced from 67% to 85%. This could make it potentially more sustainable than, or at least be comparable with lithium. Note that the inclusion of lithium is only to provide a reference value. This is a direct result from the reduced use of dispersant, increased delamination efficiency, and increase in material recovery rate [60, 61]. And the uncertainty analysis (details in the last paragraph of Methods) shows better performance of the optimized case under all scenarios when varying the parameters in Table S6 according to the Pedigree matrix. We would also like to highlight that for the molten salt methods, their related environmental impacts are mostly caused by the use of the metal monomers when synthesizing the MAX phase, as well as the use of zirconia balls during the ball‐milling process. Namely, under optimized production, the impact of mineral resource scarcity (MRS) demonstrates the most significant reduction (86% and 85% for molten‐ZnCl_2_ and molten‐CdCl_2_, respectively in Figure 6b‐e) compared with as‐designed process, which is primarily due to the optimization of zirconia balls usage during ball milling. Similar to carbon emissions, zirconia balls (under certain assumption of their lifetime) account for 91% and 90% in terms of MRS for molten‐ZnCl_2_ and molten‐CdCl_2_ methods under as‐designed production, respectively. Therefore, for molten salts synthesis, improving the reactant crushing efficiencies, developing more durable milling agents, and enhancing mass transfer dynamics should warrant significant future research. Collectively, advancing both transformative process innovations and rigorous operational optimizations across gas‐phase and molten salt synthesis routes is essential to position MXene as a leading class of high‐performance materials that can be commercially produced with low environmental footprint.

## 3 Conclusions

As a class of advanced material, MXenes have attracted significant attention due to their various advantageous properties. While the emerging gas‐phase synthesis method demonstrates relatively promising industrial potential compared to conventional etching methods that are limited by relatively higher hazardous raw materials demand, its detailed environmental impact, along with other emerging promising MXene synthesis pathways, remains to be systematically evaluated.

Therefore, we conduct a comprehensive and comparative LCA between the emerging gas‐phase and conventional molten salt etching synthesis method for scale‐up Ti_2_CCl_2_ production. From the perspective of carbon footprint, the in‐situ gas‐phase synthesis method produces MXene with relatively more optimal environmental performance, demonstrating 59% reduction compared to the molten synthesis methods under the base case. Sensitivity analysis indicates that future industrial production of Mxenes should focus on three strategies to reduce its carbon emissions,

1) improving reaction conversion rates through rational design of reaction conditions and pathways, 2) incorporating recovery systems within the production process and enhancing the recovery rate, 3) minimizing the usage of delamination solvents. We show that ideally, if all the above targets are reached, the in‐situ gas‐phase can achieve 94% carbon emission reduction compared to conventional HF‐HCl etching, with final emissions of 27 kg CO_2_‐eq/kg. This level of carbon footprint would enable MXene stand out as an eco‐friendly material for sustainable development and functional promises.

## 4 Methods

### 4.1 Description of the Four Synthesis Methods Considered

Herein, we perform a comparative environmental impact assessment for four representative MXene synthesis methods previously reported in literature, (1) in‐situ gas‐phase synthesis [35], (2) multi‐stage gas‐phase synthesis method [34], (3) the ZnCl_2_ molten salt synthesis [62], (4) CdCl_2_ molten salt synthesis [7]. Based on the laboratory performance metrics reported above, large‐scale production processes are designed for all four methods. They introduce key coupling unit operations such as the reactant recovery and the heat exchangers, which are briefly described in Figure 1, with details given in the Notes S1–S4, Tables S7–S10, and Figures S1–S4.

The in‐situ gas‐phase synthesis method involves in‐situ activation of TiCl_4_ [35], where TiCl_4_ and Ti sponge undergo in‐situ activation to generate gaseous TiCl_3_, followed by the reaction with CH_4_ for direct Ti_2_CCl_2_ synthesis at the gaseous synthetic region. And the resulting Ti_2_CCl_2_ is delaminated through grinding and ethanol treatment.

On the other hand, the multi‐stage gas‐phase synthesis method involves a pre‐synthesis step for TiCl_3_ [34], where TiCl_4_ and H_2_ are initially reacted in the first reactor. The product is subsequently condensed into a solid phase, which is then transferred to a second reactor where the controlled sublimation occurs, enabling reaction with CH_4_ to synthesize Ti_2_CCl_2_. The resulting Ti_2_CCl_2_ is further subjected to delamination via treatment with the n‐butyllithium hexane solution and additional organic solvents.

For the above two gaseous synthesis methods, we assume that inert gas argon (Ar) is employed as both a diluent gas and reactor purging medium. Heat exchange between reaction processes is implemented to optimize thermal energy management, supplemented by cooling water addition when necessary. And a gas recovery device is installed to recycle post‐reaction gases.

For the two molten salt synthesis methods (molten‐ZnCl_2_ or molten‐CdCl_2_) [7, 62], they form Ti_2_CCl_2_ through employing different solid raw chemicals, either etching Ti_2_AlC precursors in molten salt medium ZnCl_2_ or CdCl_2_. The resulting Ti_2_CCl_2_ is delaminated through the same way as multi‐stage gas‐phase synthesis method. We adopt assumptions for reactor purging, heat exchange, and reactant recycling analogous to those in gaseous synthesis methods.

### 4.2 Life Cycle Assessment (LCA)

To evaluate the scalability of the four synthesis methods while ensuring some of the laboratory‐derived parameters remain applicable, this study uses 10 kg of delaminated Ti_2_CCl_2_ per batch as the targeted scale in this study. For the LCA, a ‘from cradle to gate’ system boundary is adopted, focusing exclusively on the potential environmental impacts associated with the raw material acquisition and production stages. This scope excludes downstream use and end‐of‐life phases to eliminate uncertainties associated with specific application scenarios and waste treatment, thereby centering the analysis on the environmental burdens inherent to the four synthesis processes themselves.

The four synthesis processes are mainly divided into three main stages, namely precursor preparation, Ti_2_CCl_2_ production and Ti_2_CCl_2_ delamination (Figures S1–S4). For the precursor preparation and Ti_2_CCl_2_ production, the materials are reacted at the molar ratio provided in the literature for each method. The conversion rates are determined based on literature values (details included in Note S5), with a 10% defect introduced to account for scale‐up effects [63]. Processes for the molten salt synthesis methods are designed under the assumption that only the Ti_2_AlC precursor is produced in the precursor preparation section before the Ti_2_CCl_2_ production section. This excludes the impurities from the solid‐phase reactants to reduce computational cost in the pre‐treatment stage, so that more attention is given to the actual MXene production itself. In the Ti_2_CCl_2_ delamination stage, the dosage of the delamination solvent is determined based on the given values in the literature [34]. A 20% relative reduction in solvent dosage is consequently implemented during scale‐up based on the reaction type (details included in Note S5) [63]. Other assumptions including the inert gas consumption, cooling water utilization, and gas recovery rates are comprehensively documented in the Note S5. Additionally, for the energy balance calculations, the reaction heat of each process is a crucial parameter, as it influences the thermal management strategies and external energy demands during scaled‐up production. Therefore, this factor is inherently reflected in the energy consumption of the heating processes, which is quantitatively calculated based on the difference in the enthalpies of formation between the reactants and the products. Detailed process energy consumption calculations are included in the Note S6 and Table S11. The thermodynamic properties of the relevant materials are provided in the Note S6 and Table S12.

For each synthesis method, three scenarios are defined, which are the base case, ideal case, and conservative case. For the base case, material conservation and energy consumption parameters obtained from average values in literatures are used, whereas the most ideal and conservative parameters and assumptions are used for the other two. Unless otherwise noted, the analysis in the following sections is based on the base case. In the sensitivity analysis, a ±50% variation range was applied to all parameters to assess their impacts on total carbon emissions. The resulting change in total carbon emissions is determined for each parameter while holding others constant. Parameters causing a variation below 1% in total carbon emissions are omitted from result presentation (e.g. in the figure legends), as their negligible impacts have limited relevance for actionable decision‐making. Specially, for factors where the +50% assumptions become non‐scientific, we arbitrarily set sensitivity boundary based on reality check (e.g. maximum of 0.99 for gas recovery rate). Also, the upper boundary for the conversion rate is assumed to be the same as that reported in lab‐scale synthesis without considering the scale‐up defects.

The carbon emission is determined according to ISO 14067:2018 [64], with details as follows. The carbon emissions in the raw material acquisition stage (E_m_) are quantified by the consumption of the i th raw material (AD_mi_) and its corresponding carbon emission factor (EF_mi_). Since the carbon emission factors of most raw materials (as detailed subsequently) encompass transportation processes, no additional carbon emissions accounting is conducted for the transportation, thereby allowing focused attention on the inherent carbon emissions of four production methods for MXene. The carbon emissions in the production stage (E_p_) are derived from the carbon emissions generated by energy consumption of the designed processes. The carbon emissions associated with electricity consumption are determined by the power consumption (AD_e_) of the processes and the annual average power supply emission factor (EF_e_) of the power grid. The carbon emissions from heating account for merely 1–12% of the overall emissions in electricity‐exclusive energy source, meaning that the shift to natural gas heating has a marginal impact on the overall carbon footprint. And overall, the calculation of carbon emissions (E_GW_) can be summarized by the following Equation (1), with details listed in Notes S1–S6.

(1) $$E_{GW}=E_{m}+E_{p}=\sum_{i}\left(AD_{mi}*EF_{mi}\right)+AD_{e}*EF_{e}$$

The corresponding carbon emission factors of raw material are determined from database including literature [65, 66], Greet, and Ecoinvent [67, 68], which aggregate greenhouse gas emissions into CO_2_ equivalent (CO_2_‐eq) units 1) For the literature values, the carbon emission factor is based on the reported carbon emissions associated with the material usage. The carbon emission factors employed here exclude contributions from transportation processes, which are analyzed as a separate component in literatures. 2) For the Greet database, the carbon emission factor encompasses the entire production lifecycle of materials. For example, the carbon emissions related to titanium sponge include processes such as ore mining and dressing, titanium slag smelting, titanium chlorination and refining, titanium reduction and distillation. The carbon emission factors of certain substances (e.g. H_2_, NaCl and KCl) incorporate the contributions of transportation processes, with their corresponding items being selected for the subsequent analysis. 3) For the Ecoinvent database, the carbon emission factor is derived from corresponding process item of materials. All data is selected as global (GLO), China (CN) or rest of the world (RoW), with the ‘Allocation at the point of substitution’ (APOS) and the ‘market for’ model, and the carbon emissions are analyzed using the IPCC 2013 GWP 100a method. The application of the ‘market for’ model enables that all carbon emission factors integrate the contribution of transportation processes. For instance, the carbon emission factor of TiCl_4_ is determined by the ‘Titanium tetrachloride {GLO}| market for| APOS’ item. The detailed Ecoinvent datasets employed are listed in Table S6. The specific values of carbon emission factor are listed in the Table S13.

For the LTMS and CS methods, the overall carbon emissions are determined as the sum of the emissions from three stages (MAX phase synthesis, etching and delamination). Specifically, the emissions for the MAX phase synthesis and delamination steps are adopted from established Ti_3_C_2_T_x_ literature profiles to ensure baseline consistency [33]. Since these two methods rely on molten salt etching processes, the emissions for the etching stage are calculated by adjusting the molten‐ZnCl_2_ and molten‐CdCl_2_ models: 1) An average baseline emission is derived by updating the operating parameters (e.g. reaction temperature, reaction time and reactant ratios) based on the literatures [31, 32]. 2) The original etchant‐related impacts are then subtracted from this baseline to obtain the non‐etchant emissions. 3) The new etchant (e.g. NH_4_HF_2_, CuCl_2_, CuBr_2_) emissions are determined proportionally, using their specific molecular weights and carbon emission factors (Table S13) relative to the average baseline data. 4) For the CS method, the carbon emissions of the newly added molten salt solvent (e.g. LiBr, NaCl, KCl) during the etching process are calculated in the same manner. 5) Adding these newly calculated etchant and solvent emissions to the non‐etchant emissions yields the total carbon emissions for the etching stage.

In addition to the carbon emissions, we use the SimaPro software to conduct a comprehensive analysis of other potential environmental impact factors associated with the four methods. The life cycle inventories of the four synthesis methods are provided in Tables S14–S17. For this process, the Ecoinvent database is used together with the APOS and the ‘market for’ model [67], which is listed in Table S6. The global‐scale ReCiPe 2016 method is adopted to evaluate both at midpoint and endpoint levels, with a hierarchist (H) perspective [69]. As a representative example, the Sankey diagrams of other environmental impact factors for in‐situ gas‐phase method are presented in Figures S7–S23. To assess the terminal environment damages quantified through a single score at the endpoint level, lithium metal is introduced as a representative traditional energy material for comparative analysis with MXene.

To determine the reliability of the results, an uncertainty analysis is performed by Monte Carlo (MC) simulation. The data quality of each item in the life cycle inventory is assessed based on reliability, completeness, temporal correlation, geographical correlation, further technological correlation and sample size (Table S6). This Pedigree matrix determines the variance of lognormal distribution (σ^2^) for each inventory item. With these distributions, the MC analysis is conducted on the single score at the endpoint level by using the MC feature of SimaPro with 1000 individual runs. To avoid interpretation errors related to correlations, comparative Monte Carlo analyses are conducted between the as‐designed and optimized cases for the four synthesis methods [70].

## Author Contributions

P.P. and Q.Z. performed conceptualization. Y.H. and P.P. performed methodology. Y.H., P.P., J.W., and W.L. performed investigation. Y.H. performed data curation. M.X., F.Y., J.W., and W.L. provide resources. Y.H. and P.P. performed visualization. Y.H. and P.P. wrote the original draft. Y.H., P.P., M.X., F.Y., J.W., W.L., and Q.Z. wrote, review and edited. P.P. and Q.Z. performed funding acquisition. P.P. and Q.Z. performed project administration. P.P. and Q.Z. performed supervision.

## Conflicts of Interest

The authors declare no conflicts of interest.

## Supporting information




**Supporting File**: advs75376‐sup‐0001‐SuppMat.pdf.

## Data Availability

All data are available in the main text and the Supporting Information, and on request.

## References

[1] T. Lei , D. Wang , X. Yu , et al., “Global Iron and Steel Plant CO_2_ Emissions and Carbon‐Neutrality Pathways,” Nature 622, no. 7983 (2023): 514–520, 10.1038/s41586-023-06486-7. 37731002

[2] D. Shindell and C. J. Smith , “Climate and Air‐Quality Benefits of a Realistic Phase‐Out of Fossil Fuels,” Nature 573, no. 7774 (2019): 408–411, 10.1038/s41586-019-1554-z. 31534245

[3] H. Duan , S. Zhou , K. Jiang , et al., “Assessing China's Efforts to Pursue the 1.5°C Warming Limit,” Science 372, no. 6540 (2021): 378–385, 10.1126/science.aba8767. 33888636

[4] G. P. Wiederrecht , R. Bachelot , H. Xiong , et al., “Nanomaterials and Sustainability,” ACS Energy Letters 8, no. 8 (2023): 3443–3449, 10.1021/acsenergylett.3c01303.

[5] M. Naguib , M. Kurtoglu , V. Presser , et al., “Two‐Dimensional Nanocrystals Produced by Exfoliation of Ti_3_AlC_2_ ,” Advanced Materials 23, no. 37 (2011): 4248–4253, 10.1002/adma.201102306. 21861270

[6] D. Wang , C. Zhou , A. S. Filatov , et al., “Direct Synthesis and Chemical Vapor Deposition of 2D Carbide and Nitride MXenes,” Science 379, no. 6638 (2023): 1242–1247, 10.1126/science.add9204. 36952427

[7] V. Kamysbayev , A. S. Filatov , H. Hu , et al., “Covalent Surface Modifications and Superconductivity of Two‐Dimensional Metal Carbide MXenes,” Science 369, no. 6506 (2020): 979–983, 10.1126/science.aba8311. 32616671

[8] Y. Fan , L. Li , Y. Zhang , X. Zhang , D. Geng , and W. Hu , “Recent Advances in Growth of Transition Metal Carbides and Nitrides (MXenes) Crystals,” Advanced Functional Materials 32, no. 16 (2022): 2111357.

[9] A. VahidMohammadi , J. Rosen , and Y. Gogotsi , “The World of Two‐Dimensional Carbides and Nitrides (MXenes),” Science 372, no. 6547 (2021): 1165, 10.1126/science.abf1581. 34112665

[10] M. Naguib , V. N. Mochalin , M. W. Barsoum , and Y. Gogotsi , “25th Anniversary Article: MXenes: A New Family of Two‐Dimensional Materials,” Advanced Materials 26, no. 7 (2014): 992–1005, 10.1002/adma.201304138. 24357390

[11] C. J. Zhang , Y. Ma , X. Zhang , et al., “Two‐Dimensional Transition Metal Carbides and Nitrides (MXenes): Synthesis, Properties, and Electrochemical Energy Storage Applications,” Energy & Environmental Materials 3, no. 1 (2019): 29–55, 10.1002/eem2.12058.

[12] S. Zhang , L. Meng , Y. Hu , Z. Yuan , J. Li , and H. Liu , “Green Synthesis and Biosafety Assessment of MXene,” Small 20, no. 14 (2023): 2308600, 10.1002/smll.202308600. 37974554

[13] M. Z. Abid , K. Rafiq , A. Aslam , R. Jin , and E. Hussain , “Scope, Evaluation and Current Perspectives of MXene Synthesis Strategies for State of the Art Applications,” Journal of Materials Chemistry A 12, no. 13 (2024): 7351–7395.

[14] L. Jiang , J. Duan , J. Zhu , S. Chen , and M. Antonietti , “Iron‐Cluster‐Directed Synthesis of 2D/2D Fe–N–C/MXene Superlattice‐Like Heterostructure With Enhanced Oxygen Reduction Electrocatalysis,” ACS Nano 14, no. 2 (2020): 2436–2444, 10.1021/acsnano.9b09912. 31986009

[15] X. Li , Z. Huang , C. E. Shuck , G. Liang , Y. Gogotsi , and C. Zhi , “MXene Chemistry, Electrochemistry and Energy Storage Applications,” Nature Reviews Chemistry 6, no. 6 (2022): 389–404, 10.1038/s41570-022-00384-8. 37117426

[16] S. Palei , G. Murali , C.‐H. Kim , I. In , S.‐Y. Lee , and S.‐J. Park , “A Review on Interface Engineering of MXenes for Perovskite Solar Cells,” Nano‐Micro Letters 15, no. 1 (2023): 123, 10.1007/s40820-023-01083-9. 37160615 PMC10169986

[17] L. Wu , X. Yuan , Y. Tang , et al., “MXene Sensors Based on Optical and Electrical Sensing Signals: From Biological, Chemical, and Physical Sensing to Emerging Intelligent and Bionic Devices,” PhotoniX 4, no. 1 (2023): 1–56, 10.1186/s43074-023-00091-7.

[18] B. Li , Q.‐B. Zhu , C. Cui , et al., “Patterning of Wafer‐Scale MXene Films for High‐Performance Image Sensor Arrays,” Advanced Materials 34, no. 17 (2022): 2201298, 10.1002/adma.202201298. 35226775

[19] R. Qin , J. Nong , K. Wang , et al., “Recent Advances in Flexible Pressure Sensors Based on MXene Materials,” Advanced Materials 36, no. 24 (2024): 2312761, 10.1002/adma.202312761. 38380773

[20] M. Wei , N. Wu , B. Li , et al., “From MXene to Multimodal‐Responsive Smart, Durable Electromagnetic Interference Shielding Textiles,” Advanced Functional Materials 35 (2025): 2424312, 10.1002/adfm.202424312.

[21] Z.‐H. Zeng , N. Wu , J.‐J. Wei , et al., “Porous and Ultra‐Flexible Crosslinked MXene/Polyimide Composites for Multifunctional Electromagnetic Interference Shielding,” Nano‐Micro Letters 14, no. 1 (2022): 59, 10.1007/s40820-022-00800-0. 35138506 PMC8828842

[22] R. Verma , P. Thakur , A. Chauhan , R. Jasrotia , and A. Thakur , “A Review on MXene and its′ Composites for Electromagnetic Interference (EMI) Shielding Applications,” Carbon 208 (2023): 170–190, 10.1016/j.carbon.2023.03.050.

[23] S. Pan , J. Yin , L. Yu , et al., “2D MXene‐Integrated 3D‐Printing Scaffolds for Augmented Osteosarcoma Phototherapy and Accelerated Tissue Reconstruction,” Advanced Science 7, no. 2 (2019): 1901511, 10.1002/advs.201901511. 31993282 PMC6974945

[24] A. Maleki , M. Ghomi , N. Nikfarjam , et al., “Biomedical Applications of MXene‐Integrated Composites: Regenerative Medicine, Infection Therapy, Cancer Treatment, and Biosensing,” Advanced Functional Materials 32, no. 34 (2022): 2203430, 10.1002/adfm.202203430.

[25] H. Li , R. Fan , B. Zou , J. Yan , Q. Shi , and G. Guo , “Roles of MXenes in Biomedical Applications: Recent Developments and Prospects,” Journal of Nanobiotechnology 21, no. 1 (2023): 1–39, 10.1186/s12951-023-01809-2. 36859311 PMC9979438

[26] M. Alhabeb , K. Maleski , B. Anasori , et al., “Guidelines for Synthesis and Processing of Two‐Dimensional Titanium Carbide (Ti_3_C_2_T_x_ MXene),” Chemistry of Materials 29, no. 18 (2017): 7633–7644, 10.1021/acs.chemmater.7b02847.

[27] A. Iqbal , J. Hong , T. Y. Ko , and C. M. Koo , “Improving Oxidation Stability of 2D MXenes: Synthesis, Storage Media, and Conditions,” Nano Convergence 8, no. 1 (2021): 9, 10.1186/s40580-021-00259-6. 33723803 PMC7960843

[28] C. E. Shuck , K. Ventura‐Martinez , A. Goad , S. Uzun , M. Shekhirev , and Y. Gogotsi , “Safe Synthesis of MAX and MXene: Guidelines to Reduce Risk During Synthesis,” ACS Chemical Health & Safety 28, no. 5 (2021): 326–338, 10.1021/acs.chas.1c00051.

[29] B. Farasati Far , N. Rabiee , and S. Iravani , “Environmental Implications of Metal–Organic Frameworks and MXenes in Biomedical Applications: A Perspective,” RSC Advances 13, no. 49 (2023): 34562–34575. 38024989 10.1039/d3ra07092a PMC10668918

[30] S. F. Hansen , M. B. Nielsen , L. M. Skjolding , et al., “Maximizing the Safety and Sustainability of MXenes,” Scientific Reports 14, no. 1 (2024): 31030, 10.1038/s41598-024-82063-w. 39730668 PMC11681106

[31] H. Ding , Y. Li , M. Li , et al., “Chemical Scissor–Mediated Structural Editing of Layered Transition Metal Carbides,” Science 379, no. 6637 (2023): 1130–1135, 10.1126/science.add5901. 36927013

[32] Y. Wang , B. Zhou , Q. Tang , et al., “Ultrafast Synthesis of MXenes in Minutes via Low‐Temperature Molten Salt Etching,” Advanced Materials 36, no. 49 (2024): 2410736, 10.1002/adma.202410736. 39420679

[33] M. Dadashi Firouzjaei , S. K. Nemani , M. Sadrzadeh , E. K. Wujcik , M. Elliott , and B. Anasori , “Life‐Cycle Assessment of Ti_3_C_2_T_x_ MXene Synthesis,” Advanced Materials 35, no. 31 (2023): 2300422, 10.1002/adma.202300422. 37095074

[34] M. Xiang , Z. Shen , J. Zheng , et al., “Gas‐Phase Synthesis of Ti_2_CCl_2_ Enables an Efficient Catalyst for Lithium‐Sulfur Batteries,” The Innovation 5, no. 1 (2024): 100540, 10.1016/j.xinn.2023.100540. 38144039 PMC10746382

[35] F. Yue , M. Xiang , J. Zheng , et al., “One‐Step Gas‐Phase Syntheses of Few‐Layered Single‐Phase Ti_2_NCl_2_ and Ti_2_CCl_2_ MXenes With High Stabilities,” Nature Communications 15, no. 1 (2024): 10334, 10.1038/s41467-024-54815-9. PMC11605084 39609424

[36] A. Ungureanu , A. Sola , A. M. Ferrari , and R. Rosa , “A Systematic Review on Life Cycle Assessment (LCA) of Metal‐Organic Frameworks (MOFs) and MXenes,” Discover Chemistry 3, no. 1 (2026): 41, 10.1007/s44371-026-00495-x.

[37] V. Tzatzadakis , A. Thomos , F. Gojda , et al., “Environmental Footprint Assessment of Emerging PLA/MXene Nanocomposites,” ACS Applied Engineering Materials 4, no. 1 (2026): 157–168, 10.1021/acsaenm.5c00885.

[38] A. Ungureanu , A. Francini , P. Neri , et al., “Systematic Life Cycle Environmental Impact Comparison of Alternative Synthetic Strategies for Ti_3_C_2_T_x_ MXene,” ACS Sustainable Chemistry & Engineering 12, no. 15 (2024): 5893–5906, 10.1021/acssuschemeng.3c08491.

[39] M. Zhao , Y. Li , and S. Sun , “Analysis and Optimization of Two‐Column Cryogenic Process for Argon Recovery From Hydrogen‐Depleted Ammonia Purge Gas,” Chemical Engineering Research and Design 89, no. 7 (2011): 863–878, 10.1016/j.cherd.2010.11.014.

[40] B. Peng , C. Zhang , and J. Zhu , “Numerical Study of the Effect of the Gas and Solids Distributors on the Uniformity of the Radial Solids Concentration Distribution in CFB Risers,” Powder Technology 212, no. 1 (2011): 89–102, 10.1016/j.powtec.2011.04.036.

[41] R. Singh , P. Marchant , and S. Golczynski , “Modeling and Optimizing Gas Solid Distribution in Fluidized Beds,” Powder Technology 446 (2024): 120145, 10.1016/j.powtec.2024.120145.

[42] I. Ahmed , M. Duchesne , Y. Tan , and D. Y. Lu , “Electrically Heated Fluidized Beds─A Review,” Industrial & Engineering Chemistry Research 63, no. 10 (2024): 4205–4235, 10.1021/acs.iecr.3c04232.

[43] C. E. Shuck , M. Han , K. Maleski , et al., “MAX Phase on Structure and Properties of Resultant Ti_3_C_2_T_x_ MXene,” ACS Applied Nano Materials 2, no. 6 (2019): 3368–3376, 10.1021/acsanm.9b00286.

[44] T. S. Mathis , K. Maleski , A. Goad , et al., “Modified MAX Phase Synthesis for Environmentally Stable and Highly Conductive Ti_3_C_2_ MXene,” ACS Nano 15, no. 4 (2021): 6420–6429, 10.1021/acsnano.0c08357. 33848136

[45] C. E. Shuck , A. Sarycheva , M. Anayee , et al., “Scalable Synthesis of Ti_3_C_2_T_x_ MXene,” Advanced Engineering Materials 22, no. 3 (2020): 1901241, 10.1002/adem.201901241.

[46] S. Jolly , M. P. Paranthaman , and M. Naguib , “Synthesis of Ti_3_C_2_T_z_ MXene From Low‐Cost and Environmentally Friendly Precursors,” Materials Today Advances 10 (2021): 100139, 10.1016/j.mtadv.2021.100139.

[47] A. E. Ghazaly , H. Ahmed , A. R. Rezk , et al., “Ultrafast, One‐Step, Salt‐Solution‐Based Acoustic Synthesis of Ti_3_C_2_ MXene,” ACS Nano 15, no. 3 (2021): 4287–4293, 10.1021/acsnano.0c07242. 33635629 PMC8034768

[48] N. C. Stevens , N. G. Deen , and G. Finotello , “Hydrogen Reduction of Combusted Iron Powder: Role of the Fluidization Regime on the Conversion,” Fuel 407 (2026): 137393, 10.1016/j.fuel.2025.137393.

[49] T. Zhang , K. Shevchuk , R. J. Wang , H. Kim , J. Hourani , and Y. Gogotsi , “Delamination of Chlorine‐Terminated MXene Produced Using Molten Salt Etching,” Chemistry of Materials 36, no. 4 (2024): 1998–2006, 10.1021/acs.chemmater.3c02872.

[50] P. Huang , H. Ying , S. Zhang , and W.‐Q. Han , “Recent Advances and Perspectives of MXene Sediment: Composition, Morphology, Properties and Applications,” Coordination Chemistry Reviews 515 (2024): 215964, 10.1016/j.ccr.2024.215964.

[51] A. Inman , V. Sedajová , K. Matthews , et al., “Shear Delamination of Multilayer MXenes,” Journal of Materials Research 37, no. 22 (2022): 4006–4016, 10.1557/s43578-022-00690-3.

[52] X. Shi , Z. Yu , Z. Liu , et al., “Scalable, High‐Yield Monolayer MXene Preparation From Multilayer MXene for Many Applications,” Angewandte Chemie International Edition 64, no. 6 (2024): 202418420, 10.1002/anie.202418420. 39401092

[53] A. Inman , K. Shevchuk , M. Anayee , et al., “High‐Yield and High‐Throughput Delamination of Multilayer MXene via High‐Pressure Homogenization,” Chemical Engineering Journal 475 (2023): 146089, 10.1016/j.cej.2023.146089.

[54] A. R. Kamble , C. M. Patel , and Z. V. P. Murthy , “A Review on the Recent Advances in Mixed Matrix Membranes for Gas Separation Processes,” Renewable and Sustainable Energy Reviews 145 (2021): 111062, 10.1016/j.rser.2021.111062.

[55] M. Luberti and H. Ahn , “Review of Polybed Pressure Swing Adsorption for Hydrogen Purification,” International Journal of Hydrogen Energy 47, no. 20 (2022): 10911–10933, 10.1016/j.ijhydene.2022.01.147.

[56] Q. Zhang , K. Qiao , C. Hu , et al., “Study on Life‐Cycle Carbon Emission Factors of Electricity in China,” International Journal of Low‐Carbon Technologies 19 (2024): 2287–2298, 10.1093/ijlct/ctae181.

[57] S. Deutz and A. Bardow , “Life‐Cycle Assessment of an Industrial Direct Air Capture Process Based on Temperature–Vacuum Swing Adsorption,” Nature Energy 6, no. 2 (2021): 203–213, 10.1038/s41560-020-00771-9.

[58] V. Ntouros , I. Kousis , D. Papadaki , A. L. Pisello , and M. N. Assimakopoulos , “Life Cycle Assessment on Different Synthetic Routes of ZIF‐8 Nanomaterials,” Energies 14, no. 16 (2021): 4998, 10.3390/en14164998.

[59] J. Munuera , L. Britnell , C. Santoro , R. Cuéllar‐Franca , and C. Casiraghi , “A Review on Sustainable Production of Graphene and Related Life Cycle Assessment,” 2D Materials 9, no. 1 (2022): 012002, 10.1088/2053-1583/ac3f23.

[60] S. K. Hoekman , A. Broch , and X. Liu , “Environmental Implications of Higher Ethanol Production and Use in the U.S.: A Literature Review. Part I—Impacts on Water, Soil, and Air Quality,” Renewable and Sustainable Energy Reviews 81 (2018): 3140–3158, 10.1016/j.rser.2017.05.050.

[61] S. K. Hoekman and A. Broch , “Environmental Implications of Higher Ethanol Production and Use in the U.S.: A Literature Review. Part II—Biodiversity, Land Use Change, GHG Emissions, and Sustainability,” Renewable and Sustainable Energy Reviews 81 (2018): 3159–3177, 10.1016/j.rser.2017.05.052.

[62] M. Li , J. Lu , K. Luo , et al., “Element Replacement Approach by Reaction With Lewis Acidic Molten Salts to Synthesize Nanolaminated MAX Phases and MXenes,” Journal of the American Chemical Society 141, no. 11 (2019): 4730–4737, 10.1021/jacs.9b00574. 30821963

[63] F. Piccinno , R. Hischier , S. Seeger , and C. Som , “From Laboratory to Industrial Scale: A Scale‐Up Framework for Chemical Processes in Life Cycle Assessment Studies,” Journal of Cleaner Production 135 (2016): 1085–1097, 10.1016/j.jclepro.2016.06.164.

[64] “Greenhouse Gases — Carbon Footprint of Products — Requirements and Guidelines for Quantification,” International Organization for Standardization (ISO) , Geneva, Switzerland (2018): ISO 14067: 2018, accessed April 4, 2025, https://www.iso.org/standard/71206.html#lifecycle.

[65] M. Santiago‐Herrera , J. Ibáñez , M. De Pamphilis , et al., “Comparative Life Cycle Assessment and Cost Analysis of the Production of Ti_6_Al_4_V‐TiC Metal–Matrix Composite Powder by High‐Energy Ball Milling and Ti_6_Al_4_V Powder by Gas Atomization,” Sustainability 15, no. 8 (2023): 6649, 10.3390/su15086649.

[66] A. Heidari , E. Khaki , H. Younesi , and H. R. Lu , “Evaluation of Fast and Slow Pyrolysis Methods for Bio‐Oil and Activated Carbon Production From Eucalyptus Wastes Using a Life Cycle Assessment Approach,” Journal of Cleaner Production 241 (2019): 118394, 10.1016/j.jclepro.2019.118394.

[67] G. Wernet , C. Bauer , B. Steubing , J. Reinhard , E. Moreno‐Ruiz , and B. Weidema , “The Ecoinvent Database Version 3 (Part I): Overview and Methodology,” The International Journal of Life Cycle Assessment 21, no. 9 (2016): 1218–1230, 10.1007/s11367-016-1087-8.

[68] M. Wang , A. Elgowainy , U. Lee , et al., “Summary of Expansions and Updates in R&D Greet^®^ 2023,” Argonne National Laboratory, accessed April 4, 2023, https://publications.anl.gov/anlpubs/2023/10/185721.pdf.

[69] M. A. J. Huijbregts , Z. J. N. Steinmann , P. M. F. Elshout , et al., “ReCiPe2016: A Harmonised Life Cycle Impact Assessment Method at Midpoint and Endpoint Level,” The International Journal of Life Cycle Assessment 22, no. 2 (2017): 138–147, 10.1007/s11367-016-1246-y.

[70] G. Mark , O. Michiel , L. Jorrit , et al., “Introduction to LCA with Simapro,” PRé Sustainability (2016), report version: 5.2, accessed March 24, 2026, https://pre‐sustainability.com/files/2014/05/SimaPro8IntroductionToLCA.pdf.

