# Supplementary material for: Environmental Insights and Sustainability Opportunities for Scaled‐Up MXene Production Without Etching
Source: Adv Sci (Weinh). 2026 Apr 27;13(40):e75376. doi: 10.1002/advs.75376 (PMC13335621; doi:10.1002/advs.75376)
Supplement: Supplementary file 1 — Supporting File: advs75376‐sup‐0001‐SuppMat.pdf. [file ADVS-13-e75376-s001.pdf]

Supporting Information

Environmental Insights and Sustainability Opportunities for Scaled-up MXene Production Without Etching

Yushuai Huang, Peng Peng, Maoqiao Xiang, Fen Yue, Jiangyan Wang, Wenwei Liu, Qingshan Zhu**

This supporting information contains Supporting Figures S1-S23, Tables S1-S17, Notes 1-6.

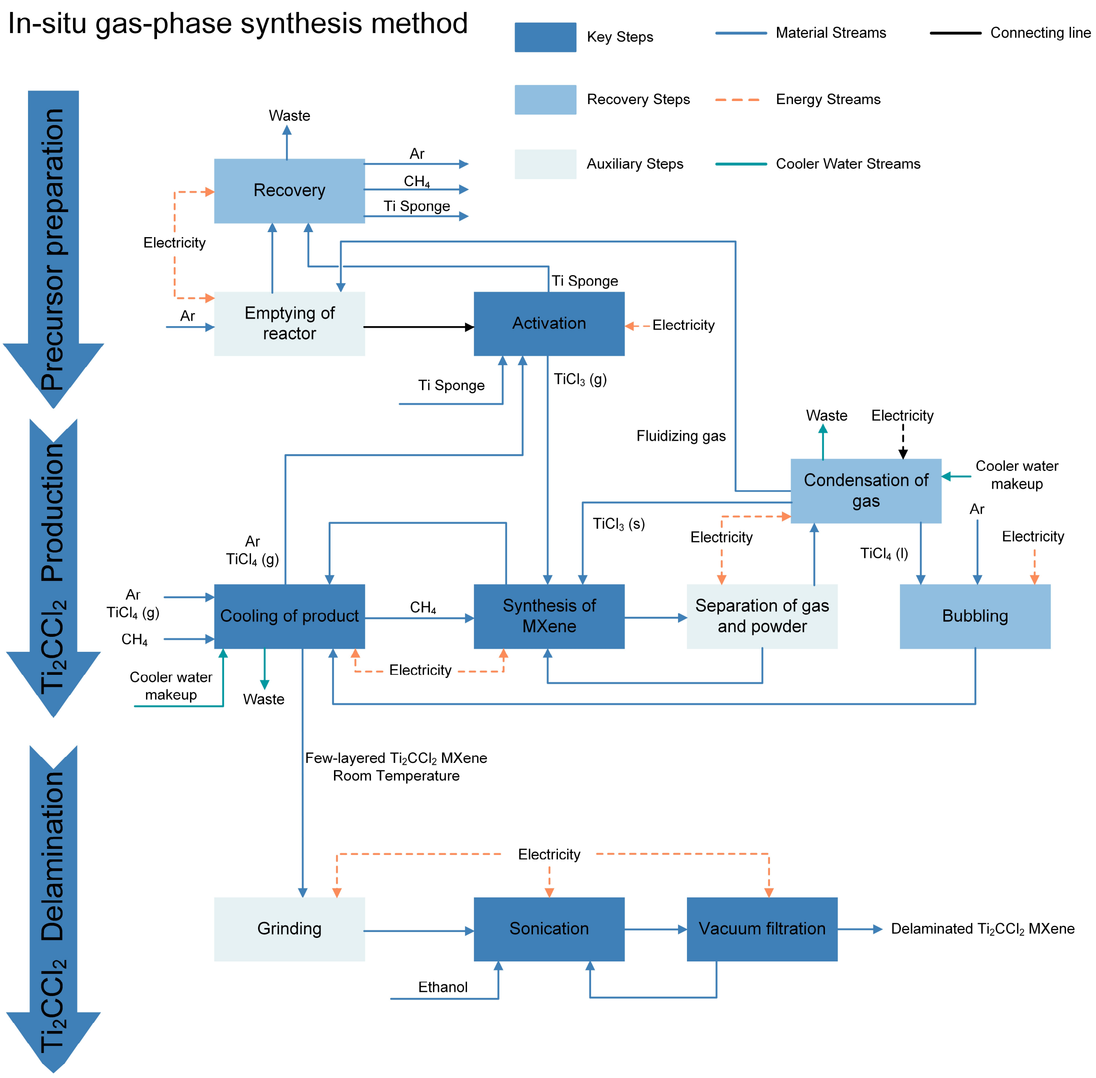

Figure S1. Flowcharts of in-situ gas-phase synthesis method.

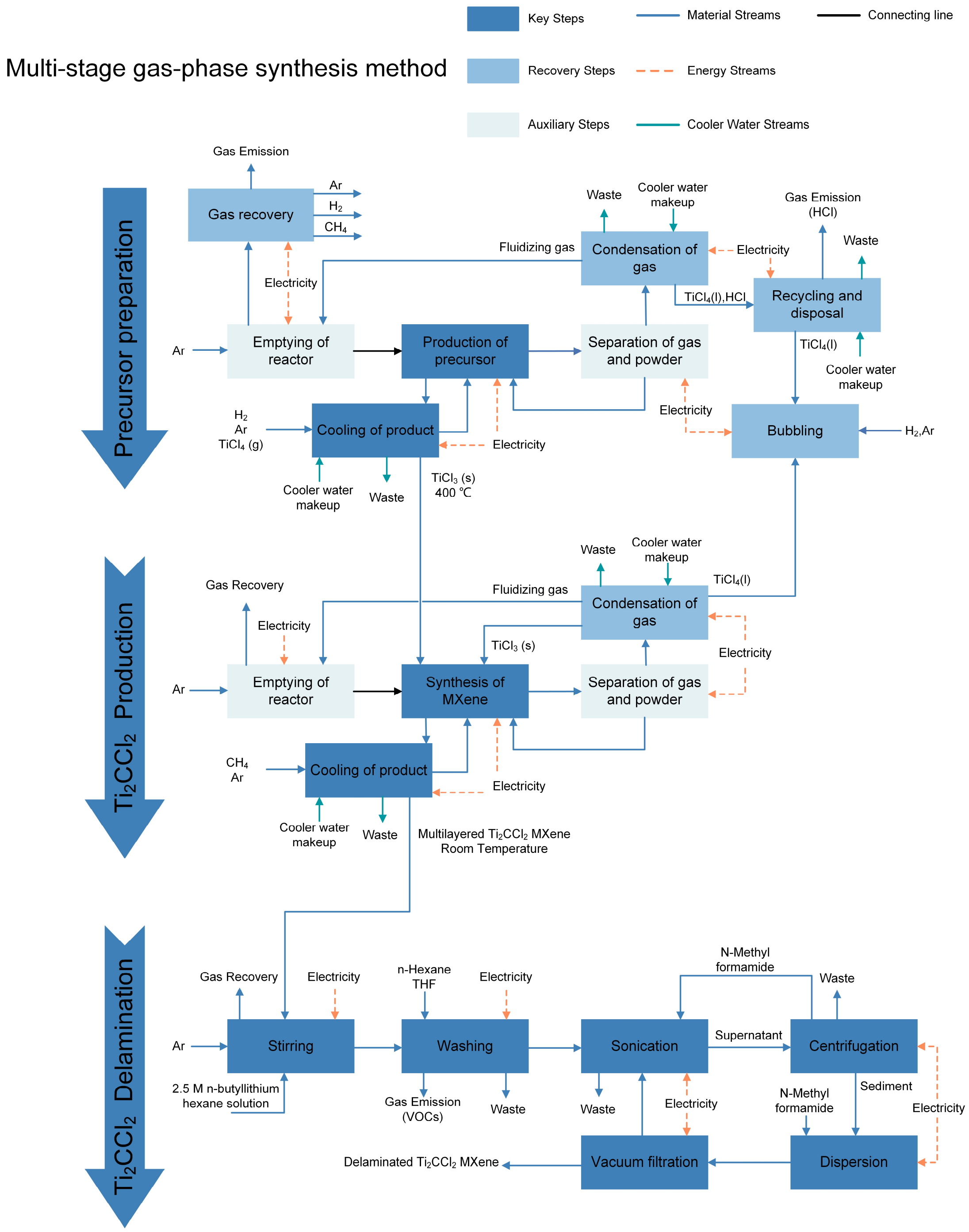

Figure S2. Flowcharts of multi-stage gas-phase synthesis method.

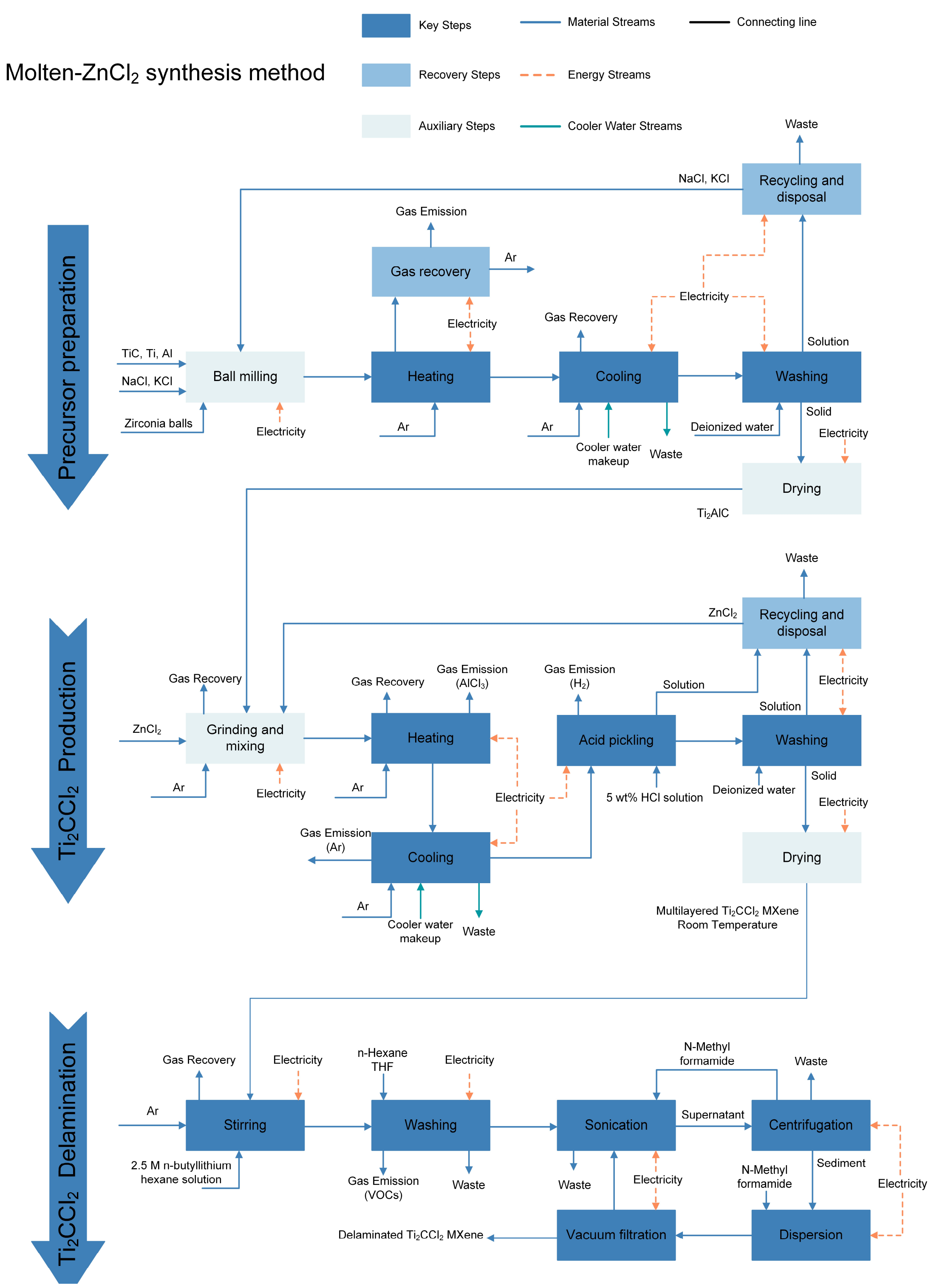

Figure S3. Flowcharts of molten-$ZnCl_2$ synthesis method.

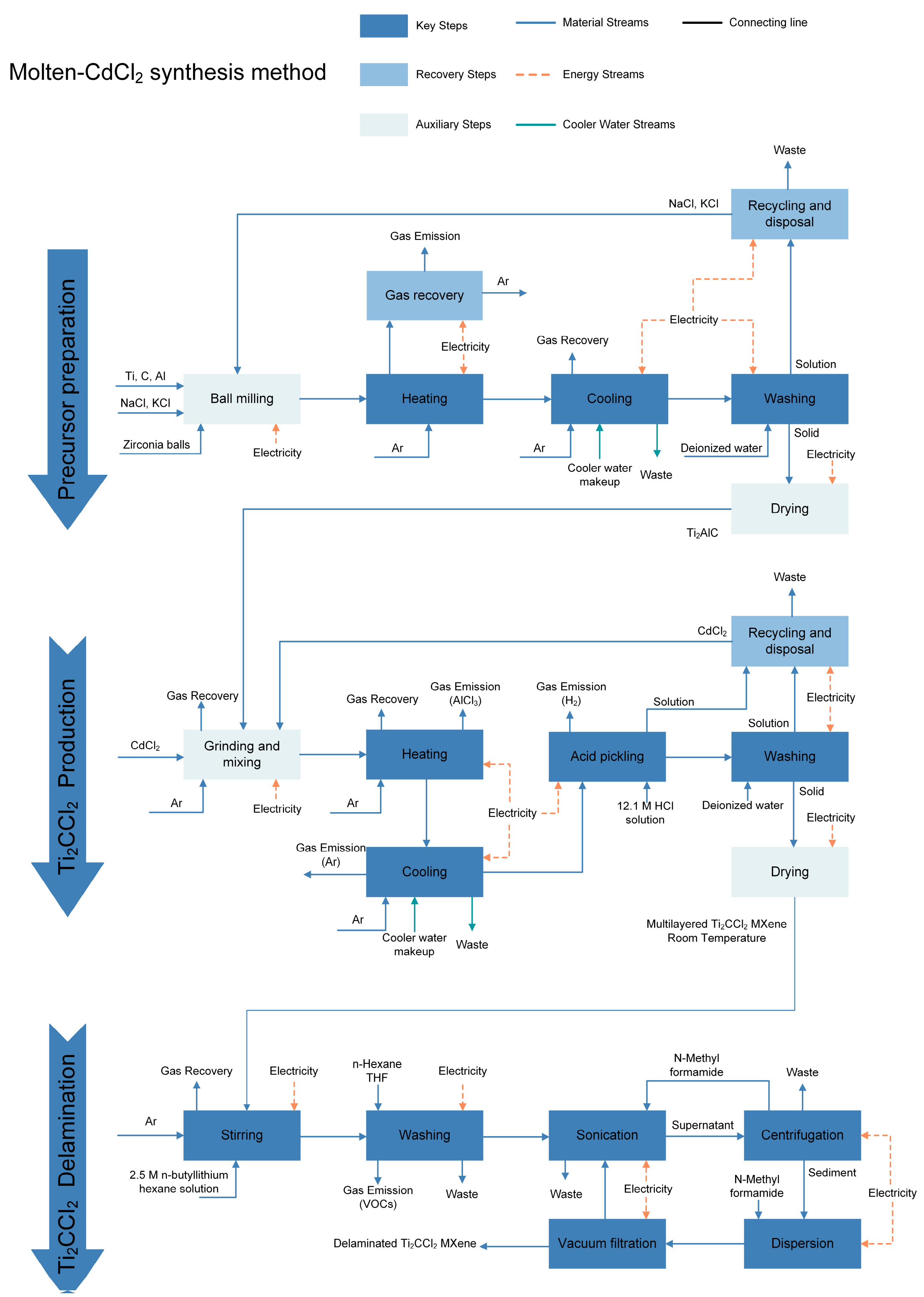

Figure S4. Flowcharts of molten-CdCl₂ synthesis method.

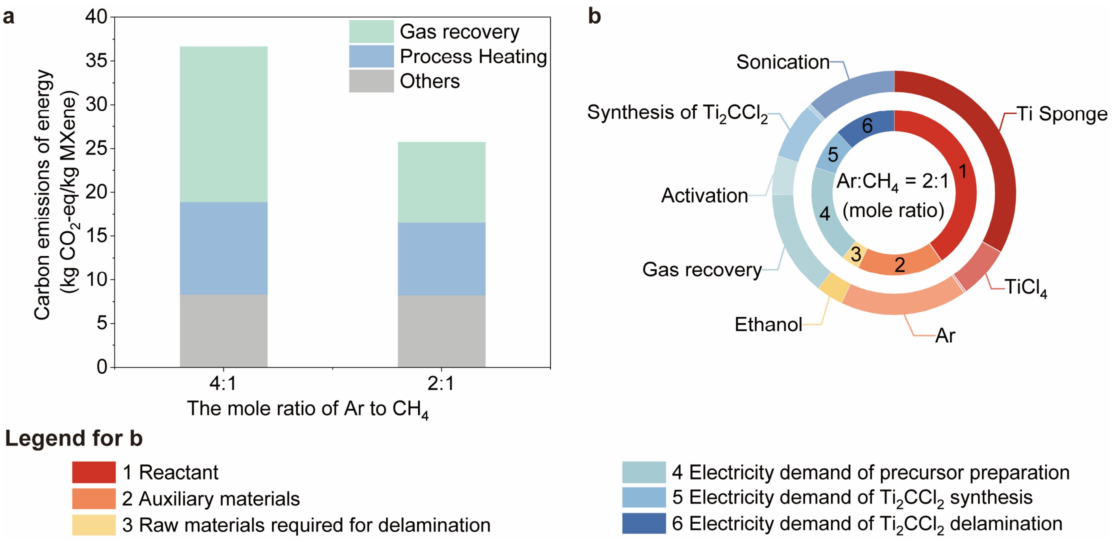

Figure S5. Carbon emissions of energy for the in-situ gas-phase synthesis method under different Ar/CH_4 mole ratios (a) Comparative results at the Ar/CH_4 mole ratios of 4:1 and 2:1. (b) Carbon emissions contributions from processes and materials at the Ar/CH_4 mole ratio of 2:1.

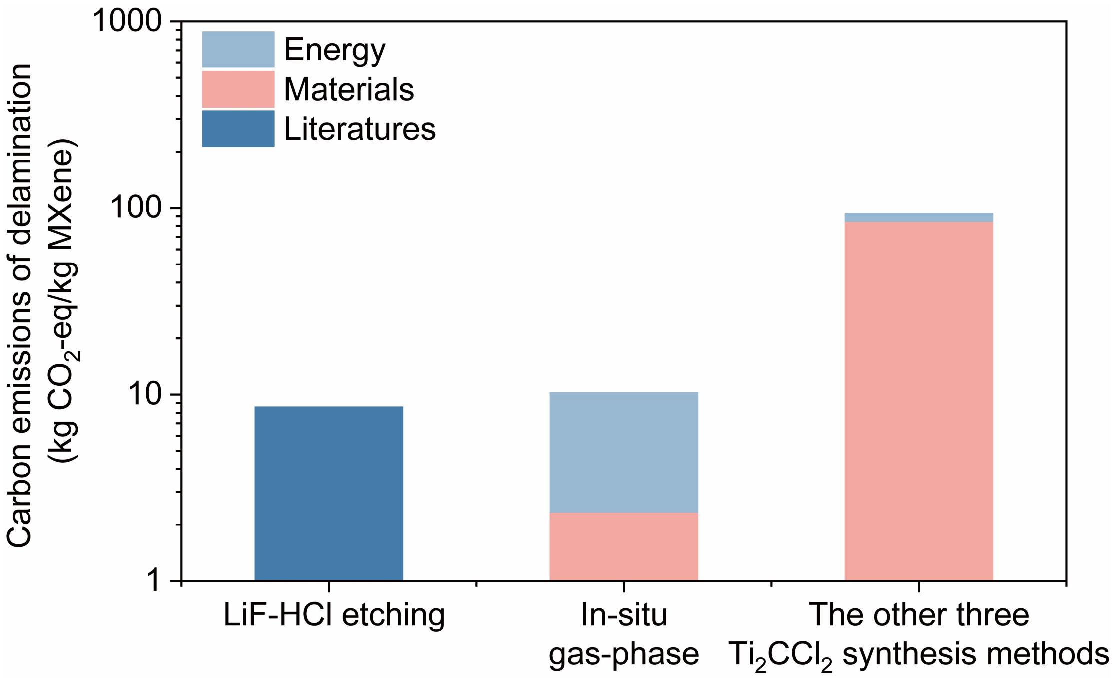

Figure S6. Comparative carbon emissions of delamination for the four Ti_2CCl_2 synthesis methods and LiF-HCl etching methods. The carbon emissions of LiF-HCl etching method are estimated from the literatures:[1, 2] Scaling factor derived from literature comparisons of identical synthesis method is applied to extrapolate small-scale results to the large-scale ones. Carbon emissions of delamination are then estimated by assuming the distributions of the single score result reflects the overall carbon emissions. The LiF-HCl etching results shown in this figure represent the average of LiF-HCl topochemical etching [3] and LiF-HCl etching of sustainable MAX[4]

in the literature.[2]

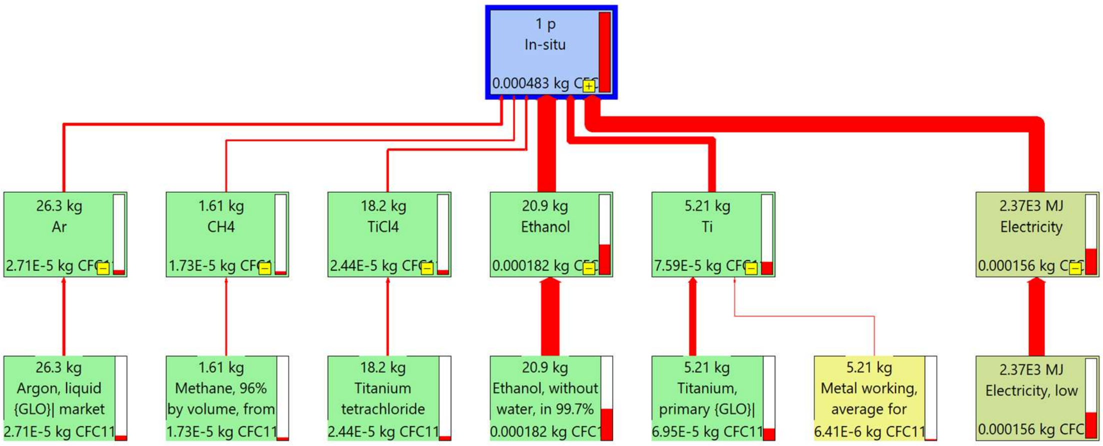

Figure S7. Sankey diagram for stratospheric ozone depletion.

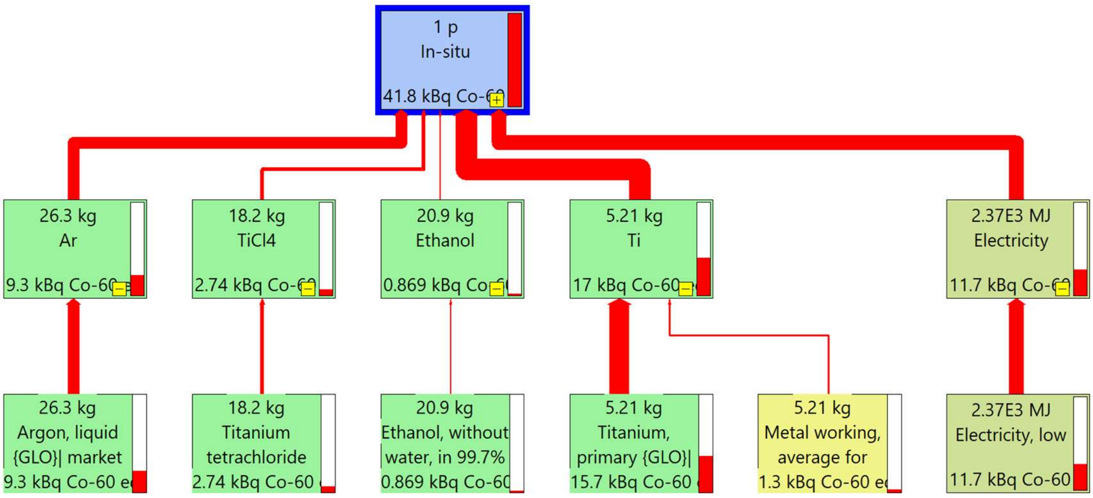

Figure S8. Sankey diagram for ionizing radiation.

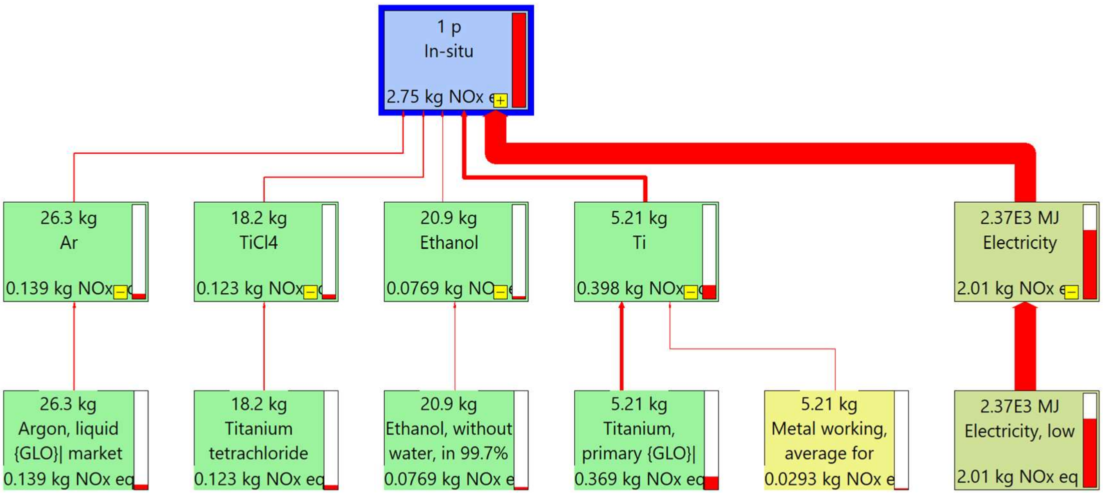

Figure S9. Sankey diagram for ozone formation-human health.

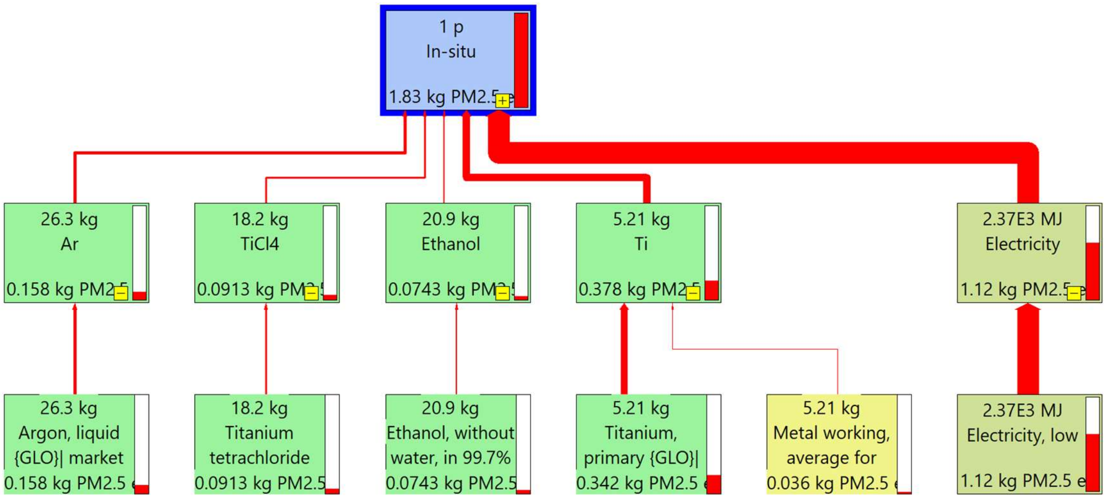

Figure S10. Sankey diagram for fine particulate matter formation.

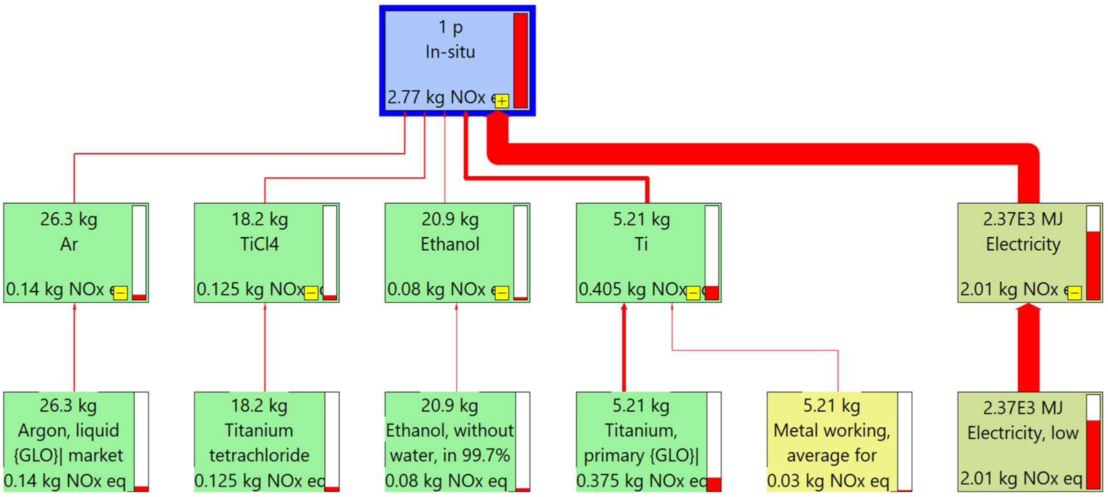

Figure S11. Sankey diagram for ozone formation-terrestrial ecosystems.

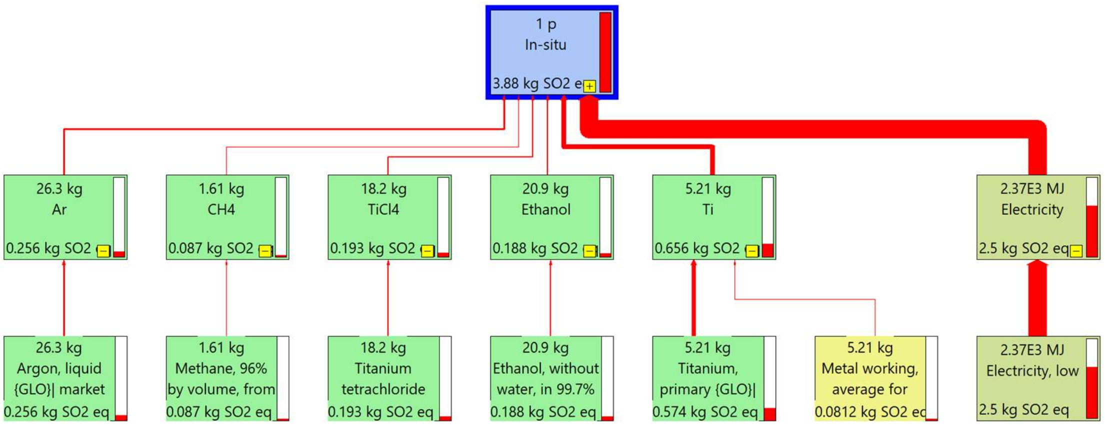

Figure S12. Sankey diagram for terrestrial acidification.

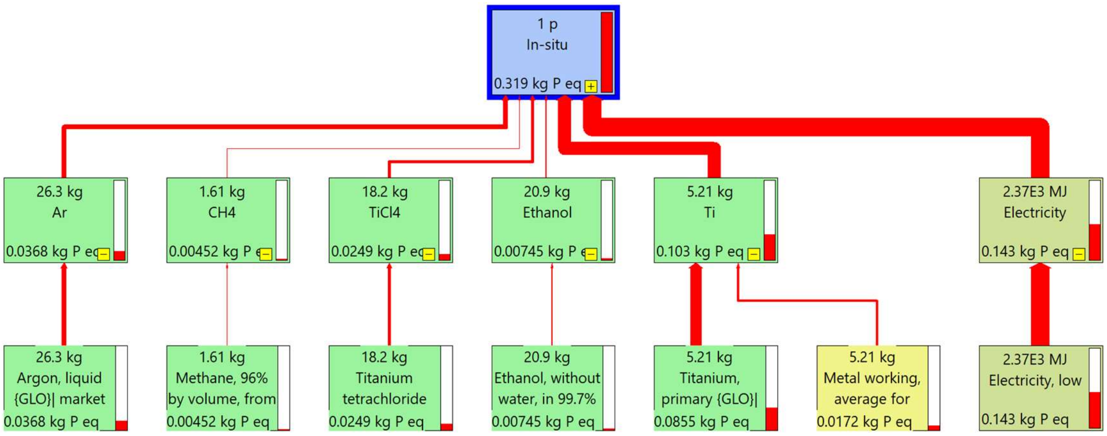

Figure S13. Sankey diagram for freshwater eutrophication.

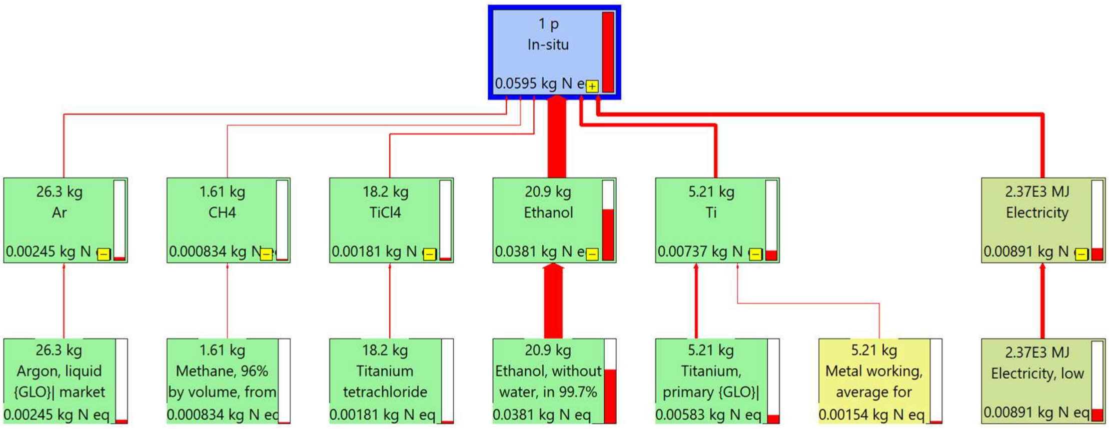

Figure S14. Sankey diagram for marine eutrophication.

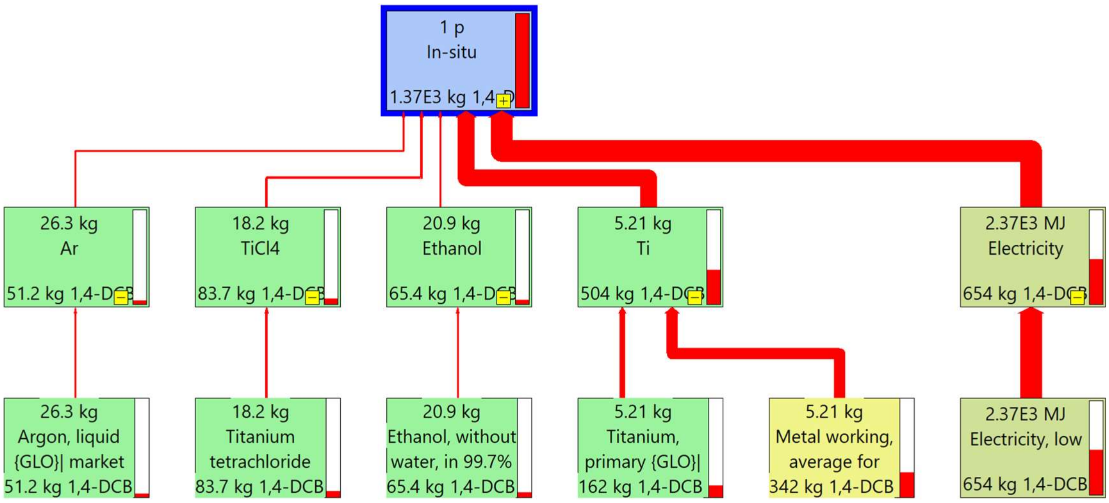

Figure S15. Sankey diagram for terrestrial ecotoxicity.

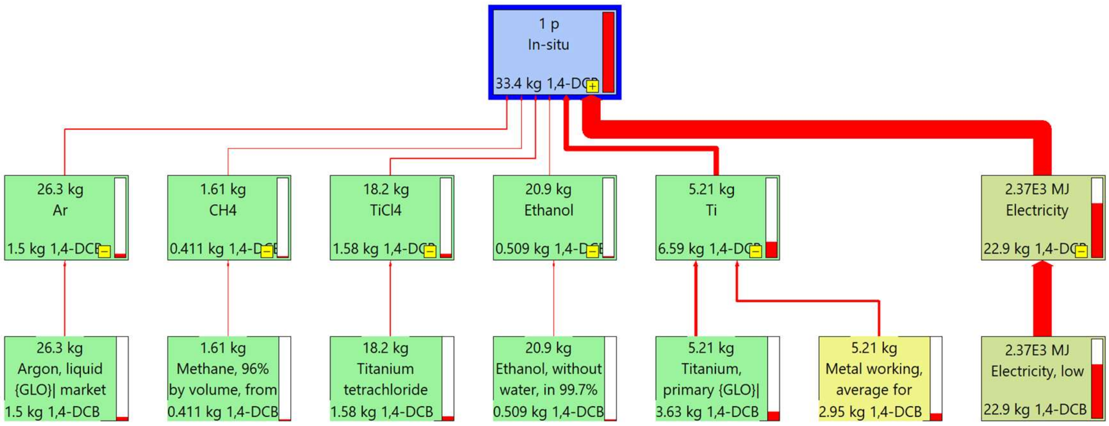

Figure S16. Sankey diagram for freshwater ecotoxicity.

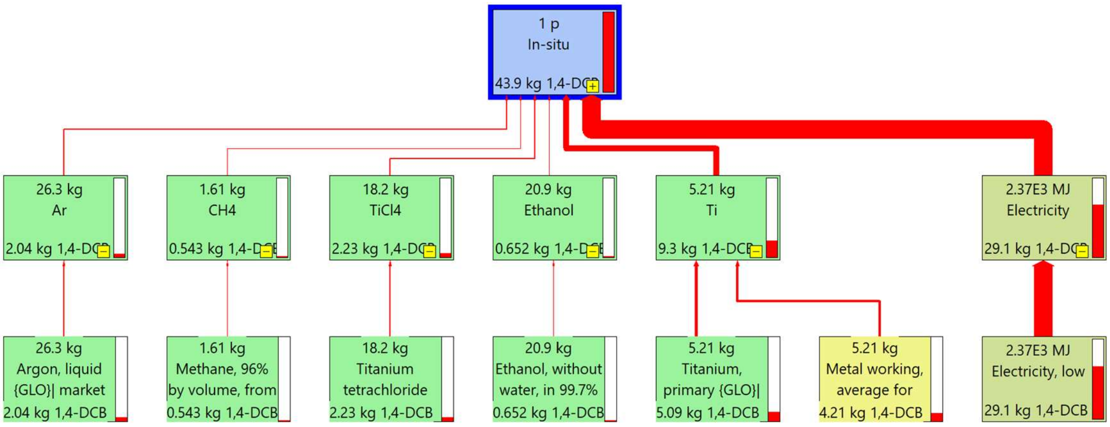

Figure S17. Sankey diagram for marine ecotoxicity.

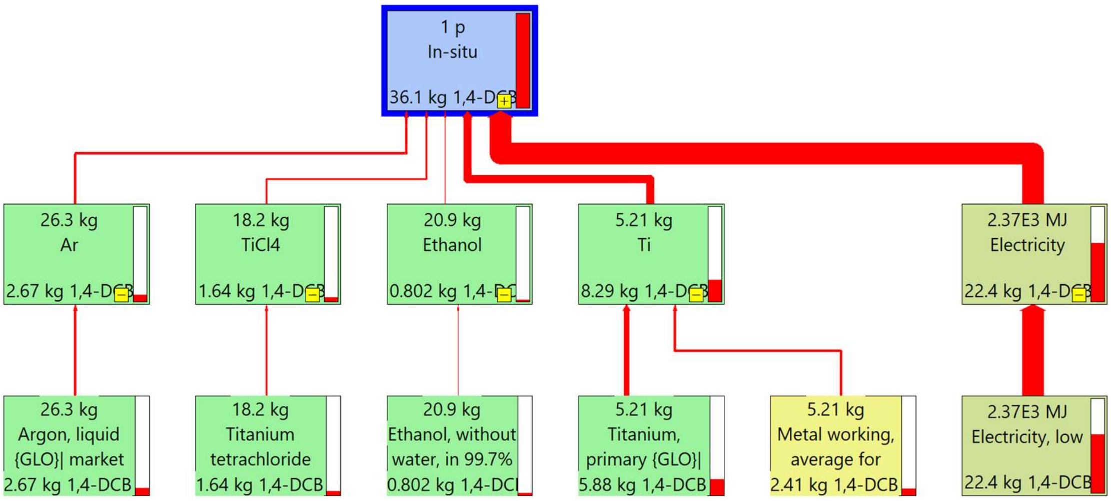

Figure S18. Sankey diagram for human carcinogenic toxicity.

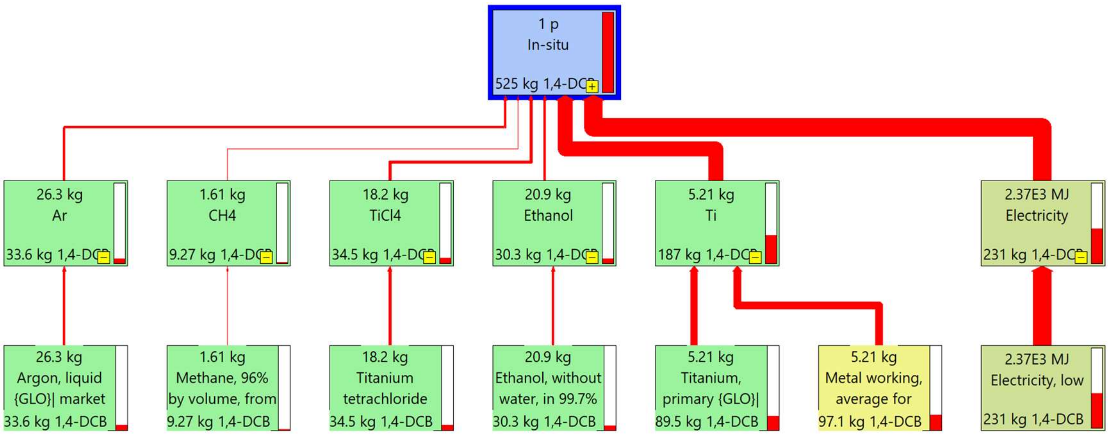

Figure S19. Sankey diagram for human non-carcinogenic toxicity.

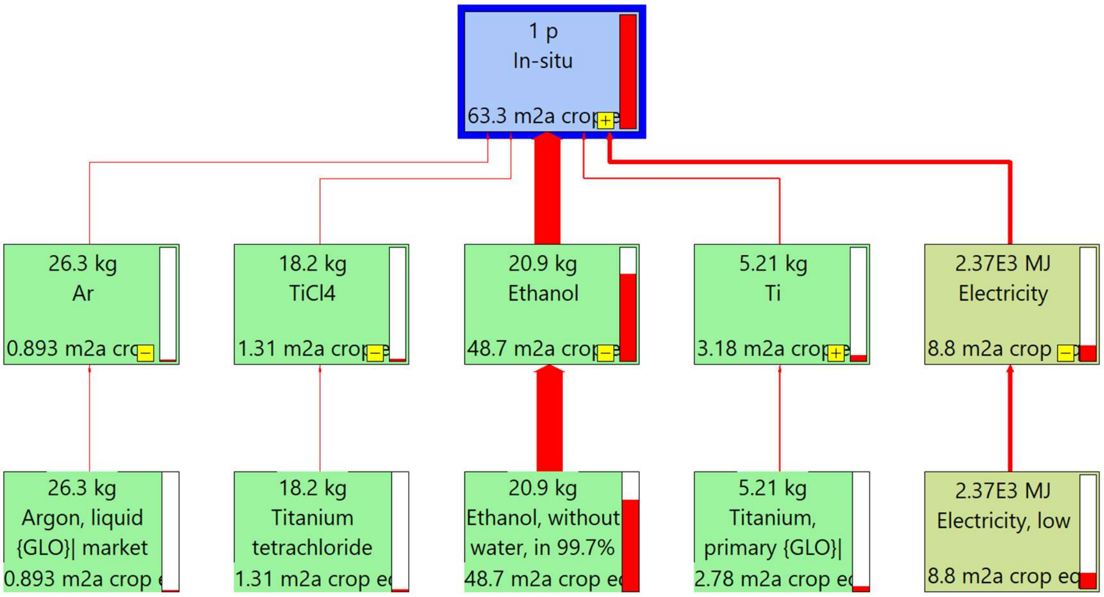

Figure S20. Sankey diagram for land use.

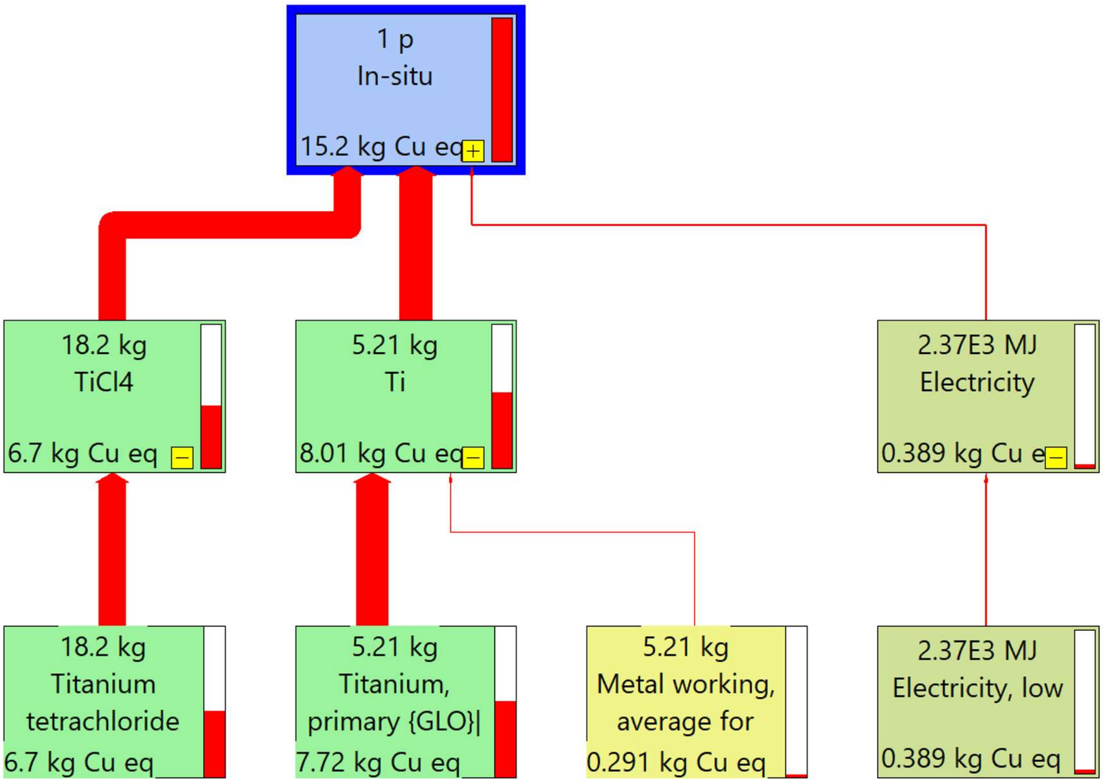

Figure S21. Sankey diagram for mineral resource scarcity.

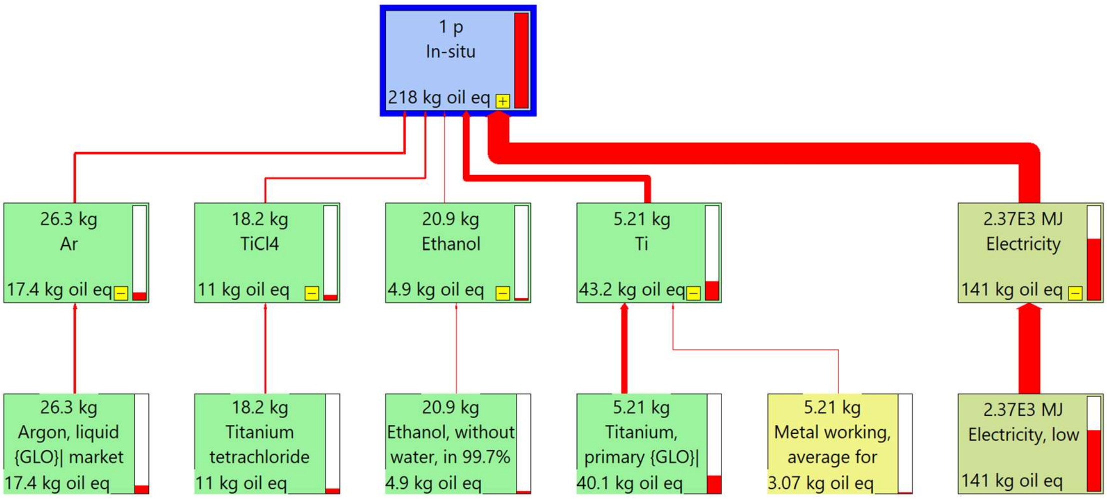

Figure S22. Sankey diagram for fossil resource scarcity.

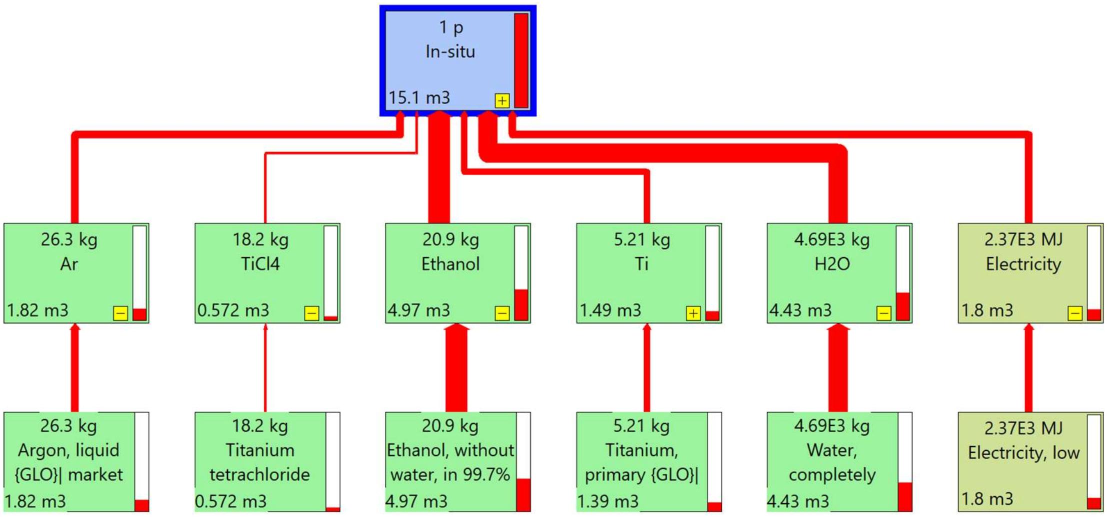

Figure S23. Sankey diagram for water consumption.

Table S1. Numerical sensitivity analysis results for in-situ gas-phase synthesis method.

Performance factors	+		-	
	Chemicals	Energy	Chemicals	Energy
Ti_2CCl_2 formation conversion rate	-15.70	-11.91	47.10	34.82
Unit dispersant usage	2.71	9.09	-0.90	-3.03
Gas recovery power	0	10.26	0	-10.26
$TiCl_3$ recovery rate	-6.72	-0.18	6.72	0.18
Sonication power	0	4.55	0	-4.55
$TiCl_3$ formation conversion rate	-0.19	-0.16	1.70	1.41
Insulation thermal conductivity	0	3.02	0	-3.02

Table S2. Numerical sensitivity analysis results for multi-stage gas-phase synthesis method.

Performance factors	+		-	
	Chemicals	Energy	Chemicals	Energy
Ti_2CCl_2 formation conversion rate	-5.73	-4.79	17.19	14.09
$TiCl_3$ formation conversion rate	-3.89	-5.46	11.67	15.95
Unit dispersant usage	17.76	4.82	-5.92	-1.61
Washing liquid amount	14.64	0	-14.64	0
NMF unit carbon emission	8.88	0	-8.88	0
Gas recovery power	0	6.61	0	-6.61
$TiCl_3$ recovery rate	-3.88	-2.60	3.88	2.58
THF:Hexane	4.14	0	-7.55	0
Sonication power	0	2.41	0	-2.41
Insulation thermal conductivity	0	2.12	0	-2.12

Table S3. Numerical sensitivity analysis results for molten-$ZnCl_2$ synthesis method.

Performance factors	+		-	
	Chemicals	Energy	Chemicals	Energy
Ball recovery rate	-7.85	0	82.38	0
Ti_2CCl_2 formation conversion rate	-11.08	-1.61	33.23	4.80
Ti_2AlC formation conversion rate	-0.81	-0.14	26.19	4.47
Unit dispersant usage	14.92	4.05	-4.97	-1.35
Washing liquid amount	12.30	0	-12.30	0
NMF unit carbon emission	7.46	0	-7.46	0
Sonication power	0	5.60	0	-5.60
Ball amount	4.81	0.02	-4.81	-0.02
THF:Hexane	3.48	0	-6.34	0
Salt recovery rate	-1.66	0	2.00	0

Table S4. Numerical sensitivity analysis results for molten-$CdCl_2$ synthesis method.

Performance factors	+		-	
	Chemicals	Energy	Chemicals	Energy
Ball recovery rate	-7.66	0	80.40	0
Ti_2CCl_2 formation conversion rate	-4.18	-0.51	36.64	4.43
Ti_2AlC formation conversion rate	-0.30	-0.04	25.35	3.07
Unit dispersant usage	14.30	3.89	-4.77	-1.30
Washing liquid amount	11.79	0	-11.79	0
NMF unit carbon emission	7.15	0	-7.15	0
Salt recovery rate	-4.65	0	5.61	0
Sonication power	0	5.24	0	-5.24
Ball amount	4.70	0.02	-4.70	-0.02
THF:Hexane	3.34	0	-6.08	0

Table S5. Uncertainty analysis results by the Monte Carlo simulation for 10 kg Ti_2CCl_2 synthesis of the four processes.

Process	Mean (Pt)	Median (Pt)	SD (Pt)	CV (%)	2.5% (Pt)	97.5% (Pt)	SEM (Pt)
In-situ gas-phase-Asd	33.81	33.82	1.65	4.88	30.67	37.19	0.05
Multi-stage gas-phase-Asd	61.17	61.11	2.85	4.65	55.78	66.93	0.09
Molten-$ZnCl_2$-Asd	85.06	85.05	3.06	3.60	79.25	90.83	0.10
Molten-$CdCl_2$-Asd	87.99	87.97	3.14	3.56	81.72	94.33	0.10
In-situ gas-phase-Opt	8.14	8.12	0.44	5.45	7.35	9.12	0.01
Multi-stage gas-phase-Opt	9.16	9.14	0.40	4.42	8.35	9.94	0.01
Molten-$ZnCl_2$- Opt	26.90	26.87	1.08	4.03	24.96	29.16	0.03
Molten-$CdCl_2$- Opt	28.90	28.92	1.14	3.95	26.59	31.14	0.04

The results are the mean, the median, the standard deviation (SD), the coefficient of variation (CV), the 2.5th percentile (2.5%), the 97.5th percentile (97.5%) and the standard error of the mean (SEM).

Table S6. The Ecoinvent datasets for the inventory.

Description	Ecoinvent dataset	Pedigree matrix**
Input		
Ti	Titanium, primary {GLO}| market for | APOS, S<br>Metal working, average for metal product manufacturing {GLO}| market for | APOS, S	(2, 1, 3, 2, 2, na), σ^2 =1.14,<br>(2, 1, 3, 2, 4, na), σ^2 =1.52
$TiCl_4$	Titanium tetrachloride {GLO}| market for | APOS, S	(2, 1, 3, 2, 2, na), σ^2 =1.14
CH_4	Methane, 96% by volume, from biogas, from high pressure network, at service station {RoW}| market for methane, 96% by volume, from biogas, from high pressure network, at service station | APOS, S	(2, 1, 3, 2, 3, na), σ^2 =1.24
H_2	Hydrogen, liquid {RoW}| market for | APOS, S	(2, 1, 3, 2, 3, na), σ^2 =1.24
TiC*	Titanium dioxide {RoW}| market for | APOS, S	(2, 1, 3, 2, 4, na), σ^2 =1.53
Al	Aluminium, primary, ingot {Row}| market for | APOS, S<br>Metal working, avergage for aluminium product manufacturing {GLO}| market for | APOS, S	(2, 1, 3, 2, 3, na), σ^2 =1.25<br>(2, 1, 3, 2, 4, na), σ^2 =1.52
NaCl	Sodium chloride, powder {GLO}| market for | APOS, S	(2, 1, 3, 2, 2, na), σ^2 =1.14
KCl	Potassium chloride, as K2O {GLO}| market for | APOS, S	(2, 1, 3, 2, 3, na), σ^2 =1.24
$ZnCl_2$*	Zinc oxide {GLO}| market for | APOS, S	(2, 1, 3, 2, 4, na), σ^2 =1.53
C	Carbon black {GLO}| market for | APOS, S	(2, 1, 3, 2, 3, na), σ^2 =1.25
$CdCl_2$	Cadmium chloride, semiconductor-grade {GLO}| market for | APOS, S	(2, 1, 3, 2, 3, na), σ^2 =1.25
Ethanol	Ethanol, without water, in 99.7% solution state, from fermentation {GLO}| market for | APOS, S	(2, 1, 3, 2, 3, na), σ^2 =1.24
n-Butyllithium hexane*	Hexane {GLO}| market for | APOS, S	(2, 1, 3, 2, 4, na), σ^2 =1.53
n-Hexane	Hexane {GLO}| market for | APOS, S	(2, 1, 3, 2, 2, na), σ^2 =1.14
THF	Tetrahydrofuran {GLO}| market for | APOS, S	(2, 1, 3, 2, 2, na), σ^2 =1.14
NMF*	Hexane {GLO}| market for | APOS, S	(2, 1, 3, 2, 4, na), σ^2 =1.53
Ar	Argon, liquid {GLO}| market for | APOS, S	(2, 1, 3, 2, 3, na), σ^2 =1.24
HCl	Hydrochloric acid, without water, in 30% solution state {RoW}| market for | APOS, S	(2, 1, 3, 2, 2, na), σ^2 =1.14
Zirconia ball	Zirconium oxide {GLO}| market for | APOS, S	(2, 1, 3, 2, 2, na), σ^2 =1.14

H_2O	Water, completely softened, from decarbonised water, at user {GLO}| market for | APOS, S	(2, 1, 3, 2, 3, na), σ^2 =1.24
Deionized water	Water, deionised, from tap water, at user {RoW}| market for water, deionised, from tap water, at user | APOS, S	(2, 1, 3, 2, 2, na), σ^2 =1.14
Electricity	Electricity, low voltage {CN}| market group for | APOS, S	(2, 1, 3, 1, 2, na), σ^2 =1.14
Output		
Ar	Argon, emissions to air	(2, 1, 3, 2, 3, na), σ^2 =1.24
$AlCl_3$	Aluminum compounds, unspecified, emissions to air	(2, 1, 3, 2, 4, na), σ^2 =1.53
CH_4	Methane, emissions to air	(2, 1, 3, 2, 3, na), σ^2 =1.24
Ethanol	Ethanol, emissions to water	(2, 1, 3, 2, 3, na), σ^2 =1.24
H_2	Hydrogen, emissions to air	(2, 1, 3, 2, 3, na), σ^2 =1.24
Waste water	Waste water, emissions to water	(2, 1, 3, 2, 3, na), σ^2 =1.24
HCl (gas-phas methods)	Hydrogen chloride, emissions to air	(2, 1, 3, 2, 3, na), σ^2 =1.24
HCl (molten methods)	Hydrogen chloride, emissions to water	(2, 1, 3, 2, 3, na), σ^2 =1.24
Metal-related waste	Chemicals, unspecified, emissions to soil	(2, 1, 3, 2, 4, na), σ^2 =1.52
Ti	Titanium, emissions to soil	(2, 1, 3, 2, 3, na), σ^2 =1.24
$TiCl_3$	Chemicals, unspecified, emissions to soil	(2, 1, 3, 2, 4, na), σ^2 =1.53
$TiCl_4$	Titanium tetrachloride, emissions to air	(2, 1, 3, 2, 3, na), σ^2 =1.24
Volatile organic compounds	VOC, volatile organic compounds, unspecified origin, emissions to air	(2, 1, 3, 2, 4, na), σ^2 =1.53
Chloride salts	Chlorides, unspecified, emissions to water	(2, 1, 3, 2, 4, na), σ^2 =1.53
Organic waste	Organic compounds (unspecified), emissions to water	(2, 1, 3, 2, 4, na), σ^2 =1.53

* Since these materials are not included in the Ecoinvent, we assume that their data are taken from similar chemicals.

**The scores in parentheses correspond to the Pedigree matrix indicators (reliability, completeness, temporal correlation, geographical correlation, further technological correlation, and sample size), which determine the variances of lognormal distributions (σ^2) for each inventory item. Both these indicators and the basic uncertainties are detailed in the reference.[5]

Table S7. Key parameters in the in-situ gas-phase synthesis method.

Process	Key parameters	Base case	Reference
Emptying of reactor	Total pressure (Pa)	400	[6]
Activation	$TiCl_3$ formation conversion rate	0.8**	[7, 8]
	$TiCl_4$ preheating temperature (°C)	75	*
	Insulation thermal conductivity (W/(m*K))	0.05	[8]
Gas recovery	Gas recovery rate	0.945	[9]
Synthesis of Ti_2CCl_2	Ti_2CCl_2 formation conversion rate	0.6	[10]
Condensation of gas	COP	5	[11]
	Water temperature difference (°C)	7.5	*
	$TiCl_3$ recovery rate	0.6	*
Sonication	Ratio of dispersant to MXene (L/kg)	47**	[8, 12, 13]

*These parameters are detailed in Note 5.

**Scale-up effects are included (see details in Note 5).

Table S8. Key parameters in the multi-stage gas-phase synthesis method.

Process	Key parameters	Base case	Reference
Emptying of reactor	Total pressure (Pa)	400	[6]
Production of precursor	$TiCl_3$ formation conversion rate	0.38**	[8, 12]
	$TiCl_4$ preheating temperature (°C)	84	*
	Insulation thermal conductivity (W/(m*K))	0.05	[8]
Recycling and disposal	Degassing temperature (°C)	40	*
Gas recovery	Gas recovery rate	0.945	[9]
Synthesis of Ti_2CCl_2	Ti_2CCl_2 formation conversion rate	0.6	[10]
Condensation of gas	COP	5	[11]
	Water temperature difference (°C)	7.5	*
	$TiCl_3$ recovery rate	0.6	*
Stirring	Impeller diameter (m)	0.2	[8]
	Agitator rotational speed (s^{-1})	3.2	[8]
	Impeller power number	0.79	[8]
Washing	THF:Hexane	1.4	*
	Washing liquid amount	1.75	*
Dispersion	Ratio of dispersant to MXene (L/kg)	47**	[8, 12, 13]

*These parameters are detailed in Note 5.

**Scale-up effects are included (see details in Note 5).

Table S9. Key parameters in the molten-$ZnCl_2$ synthesis method.

Process	Key parameter	Base case	Reference
Ball milling	Ball-powder mass ratio	12	[14]
	Ball recovery rate	0.945	*
Heating	Ti_2AlC formation conversion rate	0.97	[15]
	Ti_2CCl_2 formation conversion rate	0.57**	[8, 16]
	Reactor void fraction	0.5	*
	Insulation thermal conductivity (W/(m*K))	0.05	[8]
Cooling	COP	5	[11]
	Water temperature difference (°C)	7.5	*
Washing	Washing liquid margin	0.2	*
Recycling and disposal	Salt recovery rate	0.7	*
Drying	Water amount	0.1	*
	drying efficiency	0.8	[8]
Gas recovery	Gas recovery rate	0.945	[9]
Acid pickling	Reaction heat (kJ/mol)	-139.4	[17]
Stirring	Impeller diameter (m)	0.2	[8]
	Agitator rotational speed (s^{-1})	3.2	[8]
	Impeller power number	0.79	[8]
Washing	THF:hexane	1.4	*
	Washing liquid amount	1.75	*
Dispersion	Ratio of dispersant to Mxene (L/kg)	47**	[8, 12, 13]

*These parameters are detailed in Note 5.

**Scale-up effects are included (see details in Note 5).

Table S10. Key parameters in the molten-$CdCl_2$ synthesis method.

Process	Key parameter	Base case	Reference
Ball milling	Ball-powder mass ratio	12	[14]
	Ball recovery rate	0.945	*
Heating	Ti_2AlC formation conversion rate	0.98	[15]
	Ti_2CCl_2 formation conversion rate	0.78**	[8, 13]
	Reactor void fraction	0.5	*
	Insulation thermal conductivity (W/(m*K))	0.05	[8]
Cooling	COP	5	[11]
	Water temperature difference (°C)	7.5	*
Washing	Washing liquid margin	0.2	*
Recycling and disposal	Salt recovery rate	0.7	*
Drying	Water amount	0.1	*
	drying efficiency	0.8	[8]
Gas recovery	Gas recovery rate	0.945	[9]
Acid pickling	Reaction heat (kJ/mol)	-322.4	[18]
Stirring	Impeller diameter (m)	0.2	[8]
	Agitator rotational speed (s^{-1})	3.2	[8]
	Impeller power number	0.79	[8]
Washing	THF:hexane	1.4	*
	Washing liquid amount	1.75	*
Dispersion	Ratio of dispersant to Mxene (L/kg)	47**	[8, 12, 13]

*These parameters are detailed in Note 5.

**Scale-up effects are included (see details in Note 5).

Table S11. Comparison table of process energy consumption based on direct adoption of literature values.

Process	Values	Base Case	Reference
Grinding (kWh / ton grinded material)	8-16	12	[8]
Centrifugation (kWh / ton dried material)	1-10	5.5	[8]
Vacuum filtration (kWh / ton dried material)	1-10	5.5	[8]
Separation of gas and powder (kWh / ton powder)	1-10	5.5	[8]
Sonication (kWh / liter solution)	0.15-0.3	0.3	[19]
Gas recovery (kWh / Nm^3 Ar)	1.18, 1.22, 1.27, 1.35	1.255	[9]
Molten salt recycling (kWh / m^3 evaporated water)	5-25	15	[20]

Table S12. Thermodynamic data references for relevant materials.

Material	Thermal capacity	Solubility	Enthalpy of formation	Density	Reaction heat
NaCl-KCl	[21]	[22]		[23]	
Ti_2AlC	[24]		[25]		
ZnCl	[26]	[27]	[28]		
$CdCl_2$	[29]	[30]	[18]		
Zn+HCl					[17]

Table S13. Carbon emission factor of key materials used in the synthesis processes

Materials	Carbon emission factor ($kg\ CO_2$-eq/kg)	Source
Ti Sponge	41.42	Greet[31]
$TiCl_4$	2.49	Ecoinvent[32]
CH_4	1.11	Greet
H_2	10.08	Greet
Ti powder	58.22	Greet
TiC	14.47	[33]
Al	16.11	Greet
NaCl	0.23	Greet
KCl	0.47	Greet
$ZnCl_2$	1.12	[34]
C	3.43	Greet
$CdCl_2$	2.96	Ecoinvent
Ethanol	1.12	Greet
n-Butyllithium hexane	0.65	*
n-Hexane	0.65	Greet
Tetrahydrofuran (THF)	6.39	Ecoinvent
N-methyl formamide (NMF)	0.65	*
Ar	8.04	Greet
HCl	0.92	Greet
Zirconia ball	1.73	Greet
H_2O	2.5E-5	Ecoinvent
Deionized water	1.5E-3	Ecoinvent
NH_4HF_2	1.46	Ecoinvent**
$CuBr_2$	5.84	Ecoinvent**
Cd_3P_2	3.59	Ecoinvent**
LiBr	7.85	Ecoinvent**
$CuCl_2$	5.84	Ecoinvent**
CuS	5.84	Ecoinvent**

* Since these materials are not included in the relevant databases, we assume that they have the same carbon emission factor as n-hexane

** Since these materials are not included in the relevant databases, we assume that their carbon emission factors are the average of the corresponding dataset at the bottom.

1) NH_4HF_2: Ammonium bicarbonate {GLO}| market for | APOS, S, Ammonium carbonate {GLO}| market for | APOS, S, Ammonium chloride {GLO}| market for | APOS, S

2) $CuBr_2$, $CuCl_2$, CuS: Copper carbonate {GLO}| market for | APOS, S, Copper oxide {GLO}| market for | APOS, S, Copper sulfate {GLO}| market for | APOS, S

3) Cd_3P_2: Cadmium chloride, semiconductor-grade {GLO}| market for | APOS, S, Cadmium sulfide, semiconductor-grade {GLO}| market for | APOS, S

4) LiBr: Lithium chloride {GLO}| market for | APOS, S, Lithium fluoride {GLO}| market for | APOS, S, Lithium hexafluorophosphate {GLO}| market for | APOS, S, Lithium hydroxide {GLO}| market for | APOS, S, Lithium manganese oxide {GLO}| market for | APOS, S

Table S14. The life cycle inventory of the in-situ gas phase method.

Description	Amount	Note
Input (kg)		
Ar	26.33	Molar ratio (Ar: CH_4) = 4:1<br>Recovery rate = 94.5%<br>Inertization consumption
CH_4	1.61	Molar ratio (CH_4: $TiCl_3$) = 1.5:1<br>Recovery rate = 94.5%
$TiCl_4$	18.23	Conversion rate ($TiCl_4$ to $TiCl_3$) = 80%<br>Reaction molar ratio ($TiCl_4$: $TiCl_3$) = 3:4<br>Conversion rate ($TiCl_3$ to Ti_2CCl_2) = 60%<br>Reaction molar ratio ($TiCl_4$: Ti_2CCl_2) = 4:1
Ethanol	20.90	Condensation recovery<br>47 ml/g Ti_2CCl_2<br>Recovery rate = 94.5%
Ti	5.21	Molar ratio (Ti: $TiCl_4$) = 1:3<br>Recovery rate = 94.5%
H_2O	4694.97	Water temperature difference: 7.5 ℃
Electricity (kWh)	658.16	Sum of all energy consumptions
Output (kg)		
Ar	26.33	Molar ratio (Ar: CH_4) = 4:1<br>Recovery rate = 94.5%<br>Inertization consumption
CH_4	0.70	Reaction molar ratio (CH_4: $TiCl_3$) = 1:6<br>Conversion rate ($TiCl_3$ to Ti_2CCl_2) = 60%
H_2	0.23	Reaction molar ratio (H_2: CH_4) = 2:1
Ti	0.07	Reaction molar ratio (Ti: $TiCl_4$) = 1:3
Waste water	4694.97	Cooling water waste
$TiCl_3$	13.95	$TiCl_3$ recovery rate = 60%<br>Conversion rate ($TiCl_4$ to $TiCl_3$) = 80%<br>Conversion rate ($TiCl_3$ to Ti_2CCl_2) = 60%
Organic waste	21.00	Organic compounds and Ti_2CCl_2 waste
Ti_2CCl_2	10	Conversion rate ($TiCl_3$ to Ti_2CCl_2) = 60%<br>Loss rate during delamination = 1%

Table S15. The life cycle inventory of the multi-stage gas phase method.

Description	Amount	Note
Input (kg)		
Ar	33.68	Molar ratio (Ar: H_2) = 3:5<br>Molar ratio (Ar: CH_4) = 5:2<br>Recovery rate = 94.5%<br>Inertization consumption
CH_4	1.70	Molar ratio (CH_4: $TiCl_3$) = 1.5:1<br>Recovery rate = 94.5%
H_2	0.86	Molar ratio (H_2: $TiCl_4$) = 5:1<br>Recovery rate = 94.5%
n-Hexane	41.03	Mass ratio (washing liquid: n-butyllithium hexane) = 1.75<br>Mass ratio (THF:Hexane) = 1.4
n-Butyllithium hexane	58.95	8 ml/g Ti_2CCl_2
NMF	471.07	47 ml/g Ti_2CCl_2
THF	76.20	Mass ratio (washing liquid: n-butyllithium hexane) = 1.75<br>Mass ratio (THF:Hexane) = 1.4
$TiCl_4$	40.64	Conversion rate ($TiCl_4$ to $TiCl_3$) = 38%<br>Reaction molar ratio ($TiCl_4$: $TiCl_3$) = 1:1<br>Conversion rate ($TiCl_3$ to Ti_2CCl_2) = 60%<br>Reaction molar ratio ($TiCl_4$: Ti_2CCl_2) = 4:1
H_2O	14937.34	Water temperature difference: 7.5 ℃
Electricity (kWh)	877.40	Sum of all energy consumptions
Output (kg)		
Ar	33.68	Molar ratio (Ar: H_2) = 3:5<br>Molar ratio (Ar: CH_4) = 5:2<br>Recovery rate = 94.5%<br>Inertization consumption
CH_4	0.74	Reaction molar ratio (CH_4: $TiCl_3$) = 1:6<br>Conversion rate ($TiCl_3$ to Ti_2CCl_2) = 60%
H_2	0.65	Conversion rate ($TiCl_4$ to $TiCl_3$) = 38%<br>Reaction molar ratio (H_2: $TiCl_4$) = 1:2<br>Conversion rate ($TiCl_3$ to Ti_2CCl_2) = 60%<br>Reaction molar ratio (H_2: $TiCl_3$) = 1:3
HCl	16.49	Reaction molar ratio (HCl: $TiCl_4$) = 1:1
Waste water	14937.34	Cooling water waste
$TiCl_3$	14.69	$TiCl_3$ recovery rate = 60%<br>Conversion rate ($TiCl_4$ to $TiCl_3$) = 38%<br>Conversion rate ($TiCl_3$ to Ti_2CCl_2) = 60%
Organic waste	642.02	Organic compounds and Ti_2CCl_2 waste
Volatile organic compounds	5.86	Volatilization mass rate of washing liquid = 5%
Ti_2CCl_2	10	Conversion rate ($TiCl_3$ to Ti_2CCl_2) = 60%<br>Loss rate during delamination = 6%

Table S16. The life cycle inventory of the molten-$ZnCl_2$ method.

Description	Amount	Note
Input (kg)		
TiC	6.45	Conversion rate (TiC to Ti_2AlC) = 97%
Ti	5.16	Molar ratio (Ti:TiC) = 1:1
Al	3.20	Molar ratio (Al:TiC) = 1.1:1
NaCl	7.56	Molar ratio (NaCl:TiC) = 4:1<br>Recovery rate = 70%
KCl	9.64	Molar ratio (KCl:TiC) = 4:1<br>Recovery rate = 70%
Deionized water	363.91	Washing liquid margin = 20%<br>Solubility (NaCl and KCl: water) = 0.11:1<br>Solubility ($ZnCl_2$: water) = 4.32:1
$ZnCl_2$	25.62	Molar ratio ($ZnCl_2$: Ti_2AlC) = 6:1<br>Recovery rate = 70%
HCl	260.40	Washing liquid margin = 20%<br>Reaction molar ratio (HCl:Zn) = 2:1
Zirconia ball	113.25	Mass ratio (Zirconia ball: raw powder for Ti_2AlC) = 12:1<br>Mass ratio (Zirconia ball: raw powder for Ti_2CCl_2) = 12:1<br>Recovery rate = 94.5%
Ar	0.53	Recovery rate = 94.5%<br>Inertization consumption
n-Hexane	41.03	Mass ratio (washing liquid: n-butyllithium hexane) = 1.75<br>Mass ratio (THF:Hexane) = 1.4
n-Butyllithium hexane	58.95	8 ml/g Ti_2CCl_2
NMF	471.07	47 ml/g Ti_2CCl_2
THF	76.20	Mass ratio (washing liquid: n-butyllithium hexane) = 1.75<br>Mass ratio (THF:Hexane) = 1.4
H_2O	5091.47	Water temperature difference: 7.5 ℃
Electricity (kWh)	498.38	Sum of all energy consumptions
Output (kg)		
Ar	0.53	Recovery rate = 94.5%<br>Inertization consumption
$AlCl_3$	7.94	Conversion rate (Ti_2AlC to Ti_2CCl_2) = 57%<br>Reaction molar ratio ($AlCl_3$: Ti_2AlC) = 1:1
H_2	0.30	Conversion rate (Ti_2AlC to Ti_2CCl_2) = 57%<br>Reaction molar ratio (Zn: Ti_2AlC) = 2.5:1<br>Reaction molar ratio (H_2: Zn) = 1:1
HCl	249.55	Washing liquid margin = 20%<br>Reaction molar ratio (HCl:Zn) = 2:1
Waste water	5455.38	Cooling water and deionized water waste
Metal-related waste	120.05	TiC, Ti, Al, $AlCl_3$ and Zirconia ball waste
Organic waste	642.02	Organic compounds and Ti_2CCl_2 waste
Volatile	5.86	Volatilization mass rate of washing liquid = 5%

organic compounds		
Chloride salts	42.81	NaCl, KCl and ZnCl waste
Ti_2CCl_2	10	Conversion rate (Ti_2AlC to Ti_2CCl_2) = 57%<br>Loss rate during delamination = 6%

Table S17. The life cycle inventory of the molten-$CdCl_2$ method.

Description	Amount	Note
Input (kg)		
C	0.93	Conversion rate (TiC to Ti_2AlC) = 98%
Ti	7.42	Molar ratio (Ti: C) = 2:1
Al	2.51	Molar ratio (Al: C) = 1.2:1
NaCl	5.44	Molar ratio (NaCl: Ti) = 2:1<br>Recovery rate = 70%
KCl	6.94	Molar ratio (KCl: NaCl) = 1:1<br>Recovery rate = 70%
Deionized water	332.95	Washing liquid margin = 20%<br>Solubility (NaCl and KCl: water) = 0.11:1<br>Solubility ($CdCl_2$: water) = 1.21:1
$CdCl_2$	33.70	Molar ratio ($CdCl_2$: Ti_2AlC) = 8:1<br>Recovery rate = 70%
HCl	260.40	Washing liquid margin = 20%<br>Reaction molar ratio (HCl:Cd) = 2:1
Zirconia ball	115.34	Mass ratio (Zirconia ball: raw powder for Ti_2AlC) = 12:1<br>Mass ratio (Zirconia ball: raw powder for Ti_2CCl_2) = 12:1<br>Recovery rate = 94.5%
Ar	0.60	Recovery rate = 94.5%<br>Inertization consumption
n-Hexane	41.03	Mass ratio (washing liquid: n-butyllithium hexane) = 1.75<br>Mass ratio (THF:Hexane) = 1.4
n-Butyllithium hexane	58.95	8 ml/g Ti_2CCl_2
NMF	471.07	47 ml/g Ti_2CCl_2
THF	76.20	Mass ratio (washing liquid: n-butyllithium hexane) = 1.75<br>Mass ratio (THF:Hexane) = 1.4
H_2O	5296.08	Water temperature difference: 7.5 °C
Electricity (kWh)	474.35	Sum of all energy consumptions
Output (kg)		
Ar	0.60	Recovery rate = 94.5%<br>Inertization consumption
$AlCl_3$	7.94	Conversion rate (Ti_2AlC to Ti_2CCl_2) = 78%<br>Reaction molar ratio ($AlCl_3$: Ti_2AlC) = 1:1
H_2	0.30	Conversion rate (Ti_2AlC to Ti_2CCl_2) = 57%<br>Reaction molar ratio (Cd: Ti_2AlC) = 2.5:1<br>Reaction molar ratio (H_2: Cd) = 1:1
HCl	249.55	Washing liquid margin = 20%<br>Reaction molar ratio (HCl:Cd) = 2:1
Waste water	5629.03	Cooling water and deionized water waste
Metal-related waste	118.19	Ti, C, Al, $AlCl_3$ and Zirconia ball waste
Organic waste	642.02	Organic compounds and Ti_2CCl_2 waste
Volatile	5.86	Volatilization mass rate of washing liquid = 5%

organic compounds		
Chloride salts	46.07	NaCl, KCl and CdCl waste
Ti_2CCl_2	10	Conversion rate (Ti_2AlC to Ti_2CCl_2) = 78%<br>Loss rate during delamination = 6%

Note 1. In-situ gas-phase synthesis process: For the lab-scale synthesis reported in the literature,[7] $TiCl_4$ liquid is transported into the fluidized bed reactor by Ar, undergoing in-situ activation with titanium sponge (molar ratio of $TiCl_4$/Ti = 3:1) at 1000 °C to produce precursor $TiCl_3$. The precursor further reacts with CH_4 (molar ratio of CH_4/$TiCl_3$ = 1.5:1) at 760 °C to generate Ti_2CCl_2. The resulting Ti_2CCl_2 is initially grounded, followed by ultrasonic treatment in ethanol solution and vacuum filtration to obtain the delaminated Ti_2CCl_2. The chemical reactions involved in this process are as follows:

$$3TiCl_4 + Ti = 4TiCl_3$$

$$6TiCl_3 + CH_4 = Ti_2CCl_2 + 4TiCl_4 + 2H_2$$

Based on the above steps, the design of the scale-up production process is also divided into three main stages, namely the precursor preparation, the Ti_2CCl_2 production, and Ti_2CCl_2 delamination (Figure S1). The key engineering judgments include: 1) An industrial heat exchanger network is designed so that for example, the synthesized Ti_2CCl_2 exchanges heats with the feed gas to preheat the reactant stream while simultaneously cool the Ti_2CCl_2 product. 2) To improve material and energy efficiencies, the solid powder is separated from the gas within the existing stream and recycled back to the fluidized bed reactor. Similarly, the post-reaction stream from the fluidized bed reactor is condensed to recover $TiCl_4$, employed for the emptying operation of the reactor before the reaction, and sent to the recovery process for recycling. Assumptions and process-level calculations related to material balances, reactor designs, etc., are described in detail in Supporting Note 5.

Note 2. Multi-stage gas-phase synthesis process: For the lab-scale synthesis reported in the literature,[12] the precursor $TiCl_3$ generated by the reaction between $TiCl_4$ and H_2 (molar ratio of $TiCl_4/H_2$ = 1:5) at 950 °C is first condensed into a solid phase. The resulting solid precursor is further transported to another reactor, which further reacts with CH_4 at 770 °C (molar ratio of $CH_4/TiCl_3$ = 1.5:1) to produce Ti_2CCl_2 under controlled sublimation. The synthesized Ti_2CCl_2 is immersed in 2.5 M n-butyllithium hexane solution (10 L/kg Ti_2CCl_2) under an argon atmosphere with continuous agitation. After being processed in multi-solvent (hexane/THF) wash cycles, the lithium-intercalated Ti_2CCl_2 obtained undergoes the sonication and centrifugation in the NMF solution. The collected precipitate is further dispersed in a fresh NMF solution, and finally delaminated Ti_2CCl_2 is obtained by vacuum filtration. The chemical reactions involved are:

$$2TiCl_4 + H_2 = 2TiCl_3 + 2HCl$$

$$6TiCl_3 + CH_4 = Ti_2CCl_2 + 4TiCl_4 + 2H_2$$

Based on the above steps, the design of the multi-stage gas-phase scale-up production process is distinguished by its special features, which lie in the employment of two fluidized bed reactors for Ti_2CCl_2 production along with the replacement of ethanol by other organic solvents to obtain delaminated Ti_2CCl_2 (Figure S2). Beyond the industrial heat exchanger network and the post-reaction stream recovery process being the same as those in the in-situ gas-phase synthesis process, the engineering judgments also include the employment of argon purging as a substitute for laboratory gloveboxes to

maintain an inert atmosphere during specific processing steps, such as stirring. Similarly, assumptions and process-level calculations are described in detail in Supporting Note 5.

Note 3. $ZnCl_2$ molten salt synthesis process (Molten-$ZnCl_2$): For the lab-scale synthesis reported in the literature,[16] TiC, Ti, Al, NaCl and KCl (molar ratio 1:1:1.1:4:4) are ball-milled and reacted to generate Ti_2AlC under argon atmosphere at 1100 °C for 3 hours. After completing the cooling process, the product Ti_2AlC undergoes multiple water-based wash cycles to remove NaCl and KCl. The dried Ti_2AlC is further ball-milled with $ZnCl_2$ (molar ratio of $Ti_2AlC/ZnCl_2$ = 1:6) and reacted at 550 °C for 5 hours to produce Ti_2CCl_2. Upon cooling, the generated metallic Zn is dissolved with 5 wt% HCl solution at ambient temperature, after which HCl and $ZnCl_2$ are removed from the product surface by multiple water-based wash cycles. The dried Ti_2CCl_2 is finally delaminated via the same way following the multi-stage gas-phase synthesis process. The corresponding reactions can be summarized as:

$$TiC + Al + Ti = Ti_2AlC$$

$$Ti_2AlC + 2.5ZnCl_2 = Ti_2CCl_2 + 2.5Zn + AlCl_3$$

Based on the above steps, the features in the design of the molten-$ZnCl_2$ scale-up production process for precursor preparation and Ti_2CCl_2 production are: Ti_2AlC preparation through employing solid raw materials rather than $TiCl_3$ prepared by gas-phase chemicals, Ti_2CCl_2 formation via etching MAX phase precursors within $ZnCl_2$ molten salt medium, respectively (Figure S3). Along with similar engineering

judgments to those implemented in the gas-phase synthesis processes, salt recovery processes (e.g. NaCl, KCl and $ZnCl_2$) are introduced to the scale-up design. Assumptions and process-level calculations are described in detail in Supporting Note 5.

Note 4. $CdCl_2$ molten salt synthesis process (Molten-$CdCl_2$): For the lab-scale synthesis reported in the literature,[13] the precursor Ti_2AlC is obtained by heating Ti, C, Al, NaCl and KCl (molar ratio of 2:1:1.2:4:4) at 1080 °C for 2 hours. The resulting Ti_2AlC further reacts with $CdCl_2$ (molar ratio of 1:8) at 610 °C for 6 hours to produce Ti_2CCl_2. After the reaction, the excess $CdCl_2$ and Cd are dissolved by 12.1 M HCl solution, with further multiple water-based wash cycles applied to isolate Ti_2CCl_2. The remaining steps are consistent with those of molten-$ZnCl_2$. The chemical reactions involved in this process are as follows:

$$2Ti + Al + C = Ti_2AlC$$

$$Ti_2AlC + 2.5CdCl_2 = Ti_2CCl_2 + 2.5Cd + AlCl_3$$

Based on the above steps, the design of the molten-$CdCl_2$ scale-up production process is basically the same as that of molten-$ZnCl_2$ (Figure S4). Similarly, assumptions and process-level calculations are described in detail in Supporting Note 5.

Note 5. Material conservation of the life cycle inventory.

For the $TiCl_3$ precursor preparation of gas phase synthesis methods, the conversion rates are determined based on literature values,[7, 35] with a 10% defect introduced to account

for scale-up effects.[8] To ensure we do not overestimate the environmental impacts, the conversion rate of $TiCl_3$ to Ti_2CCl_2 is set at 60% as a conservative value for the base case.[10] For the Ti_2AlC precursor preparation of molten salt synthesis methods, the conversion rate is determined according to the purity value of Ti_2AlC reported in the literature.[15] And the scale-up effects are considered when setting the conversion rate of Ti_2AlC to Ti_2CCl_2 are all taken as the given values in the literatures.[13, 16]

In the Ti_2CCl_2 delamination step, the dosage of the solvent functioned as a dispersant for delaminating is determined based on the given values in the literature.[12] A 20% relative reduction in solvent dosage is consequently implemented during scale-up.[8] For multi-stage gas-phase and molten salt synthesis methods, parameters unreported in the literature, including the quantities and volumetric ratio of hexane and THF during the washing process of lithium-intercalated Ti_2CCl_2, are assumed during scale-up, with a sensitivity analysis to assess their impacts. For the in-situ gas-phase synthesis method, we adopt the same operational values as those employed in the other three methods because of no specific values in the literature.

All four synthesis methods require argon conditions, necessitating the device emptying operation to eliminate atmospheric interference. In this study, the operation is performed using the purging inertization method, with required inert gas usage determined by the following formula:

$$Q = F * \frac{V}{t} * \ln(\frac{C_0}{C_f})$$

Key parameters included an inertization safety factor (F) of 2, emptying duration (t) of 1 hour, the volume of the device (V), initial oxygen concentration (C_0) of 21%, and target post-inertization oxygen level (C_f) of 5%. For the gas-phase synthesis methods, no supplemental inert gas is introduced when the fluidizing gas flow rate exceeds the requirement.

For continuous gas-phase production processes, the volume of the device is determined by the following formula:

$$V = Q * \tau$$

Q is the volumetric feed flow rate and τ is the space time. In this study, we assume that the reaction time reported in the literatures corresponds to the space time.

For batch processes with solid materials, the volume of the device is obtained by the following formula:

$$V = \frac{V_{materials}}{1 - \varepsilon}$$

$V_{materials}$ is the volume of the materials, and ε denotes the void fraction which is assumed to be 0.5 in this study.

The cooler water makeup is based on the principle of heat conservation, assuming the water's temperature variation of 7.5 °C (the average of 5 °C and 10 °C), followed by a

sensitivity analysis on this parameter.

For the gas-phase synthesis methods, $TiCl_4$ is introduced into the reactor by the carrier gas through bubbling, with its flow rate quantitatively described by the following equation:

$$F_T = 0.8 * \frac{P_T}{P_0 - P_T} * F_C$$

F_T is the flow rate of $TiCl_4$, P_T indicates the saturated vapor pressure at specific temperature of $TiCl_4$, P_0 denotes the pressure of the head region of the $TiCl_4$ bubbler and F_C refers to the carrier gas flow rate.[35] In order to make the theoretical value more realistic, a proportional factor of 0.8 is systematically integrated into the framework. To achieve the specified molar ratio of reactants, thermal adjustment of $TiCl_4$ is introduced to elevate P_T, with 75 °C and 84 °C for in-situ and multi-stage gas-phase synthesis methods under laboratory-scale conditions, respectively. Furthermore, for these two gas-phase synthesis methods, the gas recovery rate is estimated at 94.5% (the average of 90% and 99%) based on established literature.[9] Furthermore, for these two gas-phase synthesis methods, the $TiCl_3$ recovery rate is assumed as 60%, owing to its sensitivity to moisture and oxidation in air,[35] with a subsequent sensitivity analysis to be conducted on this parameter.

For the molten salt synthesis methods, the volume of deionized water required in the multiple water-based wash cycles for salt removal is based on their solubility (Table

S12), while the hydrochloric acid dosage is determined according to the quantity of metallic elements generated during the reaction. To ensure complete removal of impurities, a 20% safety margin is incorporated into both quantities. The quantity of zirconia ball employed during ball-milling is determined based on literature,[14] with a 94.5% recovery rate assumed consistent with that applied in gas recovery processes. Furthermore, for these two molten salt synthesis methods, the salt recovery rate is estimated at 70% (the average of 50% and 90%) due to the tendency of salts to be lost in deionized water, followed by a sensitivity analysis on this parameter.

Note 6. Energy consumption of the life cycle inventory.

The energy consumption associated with the heating process can be determined through the following equations:[8]

$$E_{heat} = \frac{Q_{material} + Q_{loss} + Q_{react}}{\eta_{heat}}$$

$$Q_{material} = C_P * m_{mix} * (T_r - T_0)$$

$$Q_{loss} = A * \frac{k_a}{S} * (T_r - T_0) * t$$

$$Q_{react} = \sum (\Delta H_{pf} - \Delta H_{rf})$$

$Q_{material}$ is the energy to reach the reaction temperature, where C_p denotes specific heat capacity, m_{mix} corresponds to the mass of heated substances, T_r indicates the reaction temperature and T_0 refers to initial or ambient temperature. The determination of the energy to compensate for the heat loss (Q_{loss}) requires the following critical parameters: the surface area of reactor (A), thermal conductivity of the insulation material (k_a),

thickness of the insulation (S), and reaction time (t). The parameter values are adopted from literature,[8] where A corresponds to the minimum surface area under a given reactor volume (the diameter to height ratio = 1), with k_a and S directly from the reported values. The reaction enthalpy (Q_{react}) depends on the enthalpy of formation differences between reactants (ΔH_{rf}) and products (ΔH_{pf}). The enthalpies of formation for Ti_2CCl_2, $TiCl_3$, $TiCl_4$ and TiC are sourced from the literature.[12] Thermodynamic properties for remaining materials can be obtained from the references listed in Table S12, with the unspecified properties derived from Aspen Plus.

Similar to the heating energy consumption, the solid drying energy consumption can be determined by the following formula:[8]

$$E_{dry} = \frac{C_{P,liq} * m_{liq} * (T_{boil} - T_0) + \Delta H_{vap} * m_{vap}}{\eta_{dry}}$$

The energy consumption in the stirring process can be described by the following correlation:[8]

$$E_{stir} = \frac{N_P * \rho_{mix} * N^3 * d^5 * t}{\eta_{stir}}$$

Influence factors are type (N_p) and diameter (d) of the impeller, the rotational velocity of stirring (N), the density of the mixture (ρ_{mix}) as well as the stirring time (t), where N_p, d and N values are from the literature.[8]

The energy consumption of blowers employed in gas transport processes can be

obtained using the following formula:

$$E_{blower} = \frac{dP * q * t}{\mu_f * \mu_b * \mu_m}$$

Key parameters included the total pressure (dP) of 400 Pa,[6] the time (t) of specific transport processes, the gas volume delivered by the blower (q), the fan efficiency (μ_f) of 0.7, the belt efficiency (μ_b) of 0.78, and the motor efficiency (μ_m) of 0.4.

The energy consumption of the liquid transport pump can be determined by the following formula:[8]

$$E_{pump} = \frac{m * g * \Delta h}{\eta_{pump}}$$

The Δh value here is twice the reactor height plus 0.2 in order to provide sufficient margin for all reactor heights.

The cooling energy consumption is determined by the coefficient of performance (COP),[11] which describes the energy performance of chillers. According to the literature,[11] COP is selected as 5 in this study.

For gas recovery processes, argon constitutes the largest proportion of total gas consumption (accounting for 97% in the in-situ gas-phase method). Therefore, we assume that the energy consumption of argon separation is adopted as a proxy for the total energy required for gas recovery, with specific value detailed in Table S11.

The energy consumption of remaining processes such as grinding, centrifugation and sonication is detailed in Table S11.

Supporting References

1 M. Dadashi Firouzjaei, S. K. Nemani, M. Sadrzadeh, et al., "Life‐Cycle Assessment of $Ti_3C_2T_x$ MXene Synthesis," *Advanced Materials* 35, no. 31 (2023): 2300422, https://doi.org/10.1002/adma.202300422.
2 A. Ungureanu, A. Francini, P. Neri, et al., "Systematic Life Cycle Environmental Impact Comparison of Alternative Synthetic Strategies for $Ti_3C_2T_x$ MXene," *ACS Sustainable Chemistry & Engineering* 12, no. 15 (2024): 5893–5906, https://doi.org/10.1021/acssuschemeng.3c08491.
3 C. E. Shuck, M. Han, K. Maleski, et al., "Effect of Ti_3AlC_2 MAX Phase on Structure and Properties of Resultant $Ti_3C_2T_x$ MXene," *ACS Applied Nano Materials* 2, no. 6 (2019): 3368–3376, https://doi.org/10.1021/acsanm.9b00286.
4 S. Jolly, M. P. Paranthaman, M. Naguib, "Synthesis of $Ti_3C_2T_z$ MXene from Low-Cost and Environmentally Friendly Precursors," *Materials Today Advances* 10, (2021): 100139, https://doi.org/10.1016/j.mtadv.2021.100139.
5 G. Mark, O. Michiel, L. Jorrit, et al., Introduction to LCA with Simapro, https://pre-sustainability.com/files/2014/05/SimaPro8IntroductionToLCA.pdf, accessed: **2026**.
6 X. Liu, X. Zhang, J. Li, et al., "Regeneration of Iron Fuel in Fluidized Beds, Part I: Defluidization Experiments and Theoretical Prediction Model," *Powder Technology* 420, (2023): 118182, https://doi.org/10.1016/j.powtec.2022.118182.
7 F. Yue, M. Xiang, J. Zheng, et al., "One-Step Gas-Phase Syntheses of Few-Layered Single-Phase Ti_2NCl_2 and Ti_2CCl_2 MXenes with High Stabilities," *Nature Communications* 15, no. 1 (2024): 10334, https://doi.org/10.1038/s41467-024-54815-9.
8 F. Piccinno, R. Hischier, S. Seeger, et al., "From Laboratory to Industrial Scale: A Scale-up Framework for Chemical Processes in Life Cycle Assessment Studies," *Journal of Cleaner Production* 135, (2016): 1085–1097, https://doi.org/10.1016/j.jclepro.2016.06.164.
9 M. Zhao, Y. Li, S. Sun, "Analysis and Optimization of Two-Column Cryogenic Process for Argon Recovery from Hydrogen-Depleted Ammonia Purge Gas," *Chemical Engineering Research and Design* 89, no. 7 (2011): 863–878, https://doi.org/10.1016/j.cherd.2010.11.014.
10 D. Wang, C. Zhou, A. S. Filatov, et al., "Direct Synthesis and Chemical Vapor Deposition of 2D Carbide and Nitride MXenes," *Science* 379, no. 6638 (2023): 1242–1247, https://doi.org/10.1126/science.add9204.
11 F. W. Yu, K. T. Chan, R. K. Y. Sit, et al., "Review of Standards for Energy

Performance of Chiller Systems Serving Commercial Buildings," *Energy Procedia* 61, (2014): 2778–2782, https://doi.org/10.1016/j.egypro.2014.12.308.

12 M. Xiang, Z. Shen, J. Zheng, et al., "Gas-Phase Synthesis of Ti_2CCl_2 Enables an Efficient Catalyst for Lithium-Sulfur Batteries," *The Innovation* 5, no. 1 (2024): 100540, https://doi.org/10.1016/j.xinn.2023.100540.

13 V. Kamysbayev, A. S. Filatov, H. Hu, et al., "Covalent Surface Modifications and Superconductivity of Two-Dimensional Metal Carbide MXenes," *Science* 369, no. 6506 (2020): 979–983, https://doi.org/doi:10.1126/science.aba8311.

14 X. Zhao, L. Shaw, "Modeling and Analysis of High-Energy Ball Milling through Attritors," *Metallurgical and Materials Transactions A* 48, no. 9 (2017): 4324–4333, https://doi.org/10.1007/s11661-017-4195-6.

15 Y. Li, H. Kong, J. Yan, et al., "Large-Scale Conformal Synthesis of One-Dimensional MAX Phases," *Nature Communications* 15, no. 1 (2024): 9275, https://doi.org/10.1038/s41467-024-53137-0.

16 M. Li, J. Lu, K. Luo, et al., "Element Replacement Approach by Reaction with Lewis Acidic Molten Salts to Synthesize Nanolaminated MAX Phases and MXenes," *Journal of the American Chemical Society* 141, no. 11 (2019): 4730–4737, https://doi.org/10.1021/jacs.9b00574.

17 D. J. Russell, D. Thomas, L. D. Hansen, "Batch Calorimetry with Solids, Liquids and Gases in Less Than 1ml Total Volume," *Thermochimica Acta* 446, no. 1-2 (2006): 161–167, https://doi.org/10.1016/j.tca.2006.02.021.

18 B. C. Shepler, K. A. Peterson, "Chemically Accurate Thermochemistry of Cadmium: An Ab Initio Study of Cd+Xy (X = H, O, Cl, Br; Y=Cl, Br)," *JOURNAL OF PHYSICAL CHEMISTRY A* 110, no. 44 (2006): 12321–12329.

19 R. Arvidsson, D. Kushnir, B. A. Sandén, et al., "Prospective Life Cycle Assessment of Graphene Production by Ultrasonication and Chemical Reduction," *Environmental Science & Technology* 48, no. 8 (2014): 4529–4536, https://doi.org/10.1021/es405338k.

20 M. Rinne, H. Elomaa, A. Porvali, et al., "Simulation-Based Life Cycle Assessment for Hydrometallurgical Recycling of Mixed LIB and NiMH Waste," *Resources, Conservation and Recycling* 170, (2021): 105586, https://doi.org/10.1016/j.resconrec.2021.105586.

21 D. Sergeev, D. Kobertz, M. Müller, "Thermodynamics of the NaCl–KCl System," *Thermochimica Acta* 606, (2015): 25–33, https://doi.org/10.1016/j.tca.2015.03.003.

22 Q. Zhu, X. Huang, "Studies on the Calculation for the Solubility of Salt-Water System Based on Aspen Plus," *Computers and Applied Chemistry* 32, no. 10 (2015): 1223–1225, https://doi.org/10.11719/com.app.chem20151016.

23 C. Agca, J. W. McMurray, "Empirical Estimation of Densities in NaCl-KCl-UCl_3 and NaCl-KCl-YCl_3 Molten Salts Using Redlich-Kister Expansion," *Chemical Engineering Science* 247, (2022): 117086, https://doi.org/10.1016/j.ces.2021.117086.

24 M. Barsoum, I. Salama, T. El-Raghy, et al., "Thermal and Electrical Properties of Nb_2AlC, (Ti, Nb)$_2$AlC and Ti_2AlC," *METALLURGICAL AND MATERIALS TRANSACTIONS A-PHYSICAL METALLURGY AND MATERIALS SCIENCE* 33, no.

9 (2002): 2775–2779.
25 Y. Pan, Z. Yang, H. Zhang, "Exploring the Structural, Phonon Dynamical, Mechanical and Thermodynamic Properties of TM_2AlC(TM=Ti, Zr and Hf) Carbides," *Diamond and Related Materials* 144, (2024): 110966, https://doi.org/10.1016/j.diamond.2024.110966.
26 C. Robelin, P. Chartrand, "Thermodynamic Evaluation and Optimization of the (NaCl + KCl + $MgCl_2$+ $CaCl_2$+ $ZnCl_2$) System," *The Journal of Chemical Thermodynamics* 43, no. 3 (2011): 377–391, https://doi.org/10.1016/j.jct.2010.10.013.
27 A. Wachter, J. Hildebrand, "Thermodynamic Properties of Solutions of Molten Lead Chloride and Zinc Chloride," *Journal of the American Chemical Society* 52, (1930): 4655–4661.
28 W.-Y. Dan, Y.-Y. Di, Y.-J. Liu, et al., "Low-Temperature Heat Capacities and Standard Molar Enthalpy of Formation of Dichloro Bis(2-Aminopyridine) Zinc (II), $ZnCl_2(C_5H_6N_2)_2$(S)," *International Journal of Thermophysics* 31, no. 11-12 (2010): 2103–2118, https://doi.org/10.1007/s10765-010-0887-5.
29 A. ALIM, P. KURTZ, W. VANVORST, "Thermodynamic Properties of Molten Mixtures of CdCl2 and PbCl2," *Journal of Chemical and Engineering Data* 13, no. 1 (1968): 24–28.
30 Y. Huang, S.-j. Ni, F. Zou, et al., "The Phase Diagrams of the $CdCl_2$-NaCl-H_2O System at 298 K," *Russian Journal of Physical Chemistry A* 88, no. 2 (2014): 243–245, https://doi.org/10.1134/s0036024414020344.
31 M. Wang, A. Elgowainy, U. Lee, et al., Summary of Expansions and Updates in R&D Greet® 2023. Argonne National Laboratory (ANL), Argonne, IL (United States): United States, **2023**; p Medium: ED; Size: 55 p.
32 G. Wernet, C. Bauer, B. Steubing, et al., "The Ecoinvent Database Version 3 (Part I): Overview and Methodology," *International Journal of Life Cycle Assessment* 21, no. 9 (2016): 1218–1230, https://doi.org/10.1007/s11367-016-1087-8.
33 M. Santiago-Herrera, J. Ibáñez, M. De Pamphilis, et al., "Comparative Life Cycle Assessment and Cost Analysis of the Production of Ti_6Al_4V-TiC Metal–Matrix Composite Powder by High-Energy Ball Milling and Ti_6Al_4V Powder by Gas Atomization," *Sustainability* 15, no. 8 (2023): 6649, https://doi.org/10.3390/su15086649.
34 A. Heidari, E. Khaki, H. Younesi, et al., "Evaluation of Fast and Slow Pyrolysis Methods for Bio-Oil and Activated Carbon Production from Eucalyptus Wastes Using a Life Cycle Assessment Approach," *Journal of Cleaner Production* 241, (2019): 118394, https://doi.org/10.1016/j.jclepro.2019.118394.
35 M. Song, Y. Yang, M. Xiang, et al., "Synthesis of Nano-Sized TiC Powders by Designing Chemical Vapor Deposition System in a Fluidized Bed Reactor," *Powder Technology* 380, (2021): 256–264, https://doi.org/10.1016/j.powtec.2020.11.045.
